# Molecular analysis of phosphomannomutase (PMM) genes reveals a unique *PMM *duplication event in diverse *Triticeae *species and the main PMM isozymes in bread wheat tissues

**DOI:** 10.1186/1471-2229-10-214

**Published:** 2010-10-05

**Authors:** Chunmei Yu, Yiwen Li, Bin Li, Xin Liu, Lifang Hao, Jing Chen, Weiqiang Qian, Shiming Li, Guanfeng Wang, Shiwei Bai, Hua Ye, Huanju Qin, Qianhua Shen, Liangbiao Chen, Aimin Zhang, Daowen Wang

**Affiliations:** 1 The State Key Laboratory of Plant Cell and Chromosome Engineering, Institute of Genetics and Developmental Biology, Chinese Academy of Sciences, Beijing 100101, PR China; 2 Life Science School, Nantong University, Nantong 226019, PR China; 3 Graduate University of Chinese Academy of Sciences, Beijing 100049, PR China

## Abstract

**Background:**

Phosphomannomutase (PMM) is an essential enzyme in eukaryotes. However, little is known about *PMM *gene and function in crop plants. Here, we report molecular evolutionary and biochemical analysis of *PMM *genes in bread wheat and related *Triticeae *species.

**Results:**

Two sets of homoeologous *PMM *genes (*TaPMM-1 *and *2*) were found in bread wheat, and two corresponding *PMM *genes were identified in the diploid progenitors of bread wheat and many other diploid *Triticeae *species. The duplication event yielding *PMM-1 *and *2 *occurred before the radiation of diploid *Triticeae *genomes. The *PMM *gene family in wheat and relatives may evolve largely under purifying selection. Among the six *TaPMM *genes, the transcript levels of *PMM-1 *members were comparatively high and their recombinant proteins were all enzymatically active. However, *PMM-2 *homoeologs exhibited lower transcript levels, two of which were also inactive. TaPMM-A1, B1 and D1 were probably the main active isozymes in bread wheat tissues. The three isozymes differed from their counterparts in barley and *Brachypodium distachyon *in being more tolerant to elevated test temperatures.

**Conclusion:**

Our work identified the genes encoding PMM isozymes in bread wheat and relatives, uncovered a unique *PMM *duplication event in diverse *Triticeae *species, and revealed the main active PMM isozymes in bread wheat tissues. The knowledge obtained here improves the understanding of *PMM *evolution in eukaryotic organisms, and may facilitate further investigations of *PMM *function in the temperature adaptability of bread wheat.

## Background

Phosphomannomutase (PMM, EC 5.4.2.8), catalyzing the interconversion between mannose-6-phosphate and mannose-1-phosphate, is an essential and conserved enzyme in eukaryotic organisms [1,2]. Mannose-1-phosphate is necessary for synthesizing the vital cellular metabolite GDP-mannose, which plays a crucial role in the formation of polysaccharide chains required for the glycosylation of protein and lipid molecules [3,4]. In higher plants, GDP-mannose also acts as an important precursor for the biosynthesis of the key antioxidant ascorbic acid (AsA) [5,6], and the mannose containing polysaccharides are essential for the development of functional cell walls [2,4]. From available information, the copy number of functional *PMM *in diploid eukaryotic species varies from one (i.e., in *Saccharomyces cerevisiae *and *Arabidopsis thaliana*) to two (e.g., in human). PMM proteins from different eukaryotic species are highly similar in primary structure, with more than 50% amino acid sequence identities found among the PMMs from *S. cerevisiae*, *Arabidopsis *and human [6]. The crystal structure and catalytic mechanism of mammalian PMMs are well understood [7], and there is also a wealth of information on human PMM2, whose point mutations lead to a genetically inherited disease CDG-Ia [8-11].

Two recent molecular genetic studies in *Arabidopsis *show that defect in *PMM *function reduces AsA biosynthesis and protein glycosylation, causing enhanced susceptibility to oxidation stress and plant death at high growth temperature (28°C) [6,12]. The requirement of a functional PMM protein for *Arabidopsis *to grow at 28°C is consistent with the pioneering finding of temperature sensitive PMM mutations that arrest *S. cerevisiae *growth at 37°C [1], suggesting that PMM may be generally important for the temperature adaptability of eukaryotes.

Compared to the above progress, little is known about *PMM *gene and function in crop plants, although broad temperature adaptability is vitally important for crops to achieve high yield potential under diverse environmental conditions. To improve our understanding on *PMM *in crop plants, we have embarked on a systematic molecular and biochemical analysis of *PMM *genes in bread wheat (*T. aestivum*, 2n = 6x = 42, AABBDD), a polyploid crop species cultivated in both temperate and tropical regions and with harvest possible under a wide range of temperature conditions [13,14]. Bread wheat belongs to the tribe *Triticeae*, which is composed of approximately 350 species, and contains both diploid (such as barley and rye) and polyploid (e.g., bread wheat) crop plants [15]. The diploid genomes in *Triticeae *species are homoeologous, and may be combined to form polyploids through natural and artificial hybridizations [16]. Bread wheat was evolved through natural hybridization between tetraploid wheat (*T. turgidum*, 2n = 4x = 28, AABB) and *Aegilops tauschii *(2n = 2x = 14, DD) about 8000 years ago [16-18]. *T. turgidum *was formed about 0.5 million years ago (MYA) through spontaneous hybridization between an unidentified *Triticeae *species containing the B genome and the wild einkorn wheat *T. urartu *(2n = 2x = 14, AA) [19-22]. The A, B and D genomes diverged from each other around 2.5 to 4.5 MYA, whereas the divergence of wheat and barley occurred around 11 MYA [19-22]. Although bread wheat and related *Triticeae *species generally contain large and complex genomes, the advent of structural and functional genomics research in model cereals (rice, *Brachypodium distachyon*) has made it possible to conduct relatively detailed molecular genetic and biochemical studies of important genes both within *Triticeae *and among rice, *Brachypodium *and *Triticeae *species. The genomic resources of rice and *B. distachyon *(e.g., molecular markers, annotated genomic information) have both been proved to be very useful in the investigations of important *Triticeae *genes, such as *Ph1 *and *Lr34/Yr18 *in common wheat and *Ppd-H1 *and *Cly1 *in barley [23-26].

From the information presented above, the main objectives of this work were to 1) determine *PMM *genes and loci in bread wheat and related *Triticeae *species, and 2) investigate the evolution of *Triticeae PMM *genes and the main active PMM isozymes in bread wheat tissues, with the aid of the genomic knowledge of rice and *B. distachyon*.

## Results

### Isolation and characterization of *PMM *genes

Two approaches (bioinformatic analysis, molecular cloning) were used to identify and isolate the *PMM *genes in *B. distachyon*, bread wheat and related *Triticeae *species (barley, *T. urartu*, *T. turgidum*, *Ae. tauschii*). In total, 17 distinct *PMM *coding sequences were isolated (Table 1). A single *PMM *gene was found for both rice (*OsPMM*) and *B. distachyon *(*BdPMM*). The cDNA of *OsPMM *was reported previously [6], whereas that of *BdPMM *was isolated in this work using RT-PCR (Table 1).

**Table 1:** *PMM *genes from *B. distachyon*, bread wheat and related *Triticeae *species, and rice ^a^

Species	Gene	gDNA (bp)	cDNA (bp)	ORF	Protein (aa)
*Brachypodium distachyon* (Bd21) ^b^	*BdPMM*	2696	750	+	249
Barley (*Hordeum vulgare*)	*HvPMM-1*	2185	750	+	249
(Betzes)	*HvPMM-2*	3263	771	-^c^	-
*Triticum urartu*	*TuPMM-A1*	2228	750	+	249
(DV877 and IE29-1)	*TuPMM-A2*	2542	751	-^d^	-
*Aegilops tauschii*	*AetPMM-D1*	2409	759	+	252
(AS67 and AS91)	*AetPMM-D2*	2348	756	+	251
*Triticeae turgidum *ssp. *durum*	*TtPMM-A1*	2227	750	+	249
(Langdon)	*TtPMM-A2*	2781	752	-^d^	-
	*TtPMM-B1*	2256	750	+	249
	*TtPMM-B2*	2500	747	+	248
Bread wheat (*Triticum aestivum*)	*TaPMM-A1*	2225	750	+	249
(Xiaoyan 54, Chinese Spring)	*TaPMM-A2*	2778	751	-^d^	-
	*TaPMM-B1*	2256	750	+	249
	*TaPMM-B2*	2500	747	+	248
	*TaPMM-D1*	2409	759	+	252
	*TaPMM-D2*	2346	756	+	251
Rice (*Oryza sativa *ssp. *japonica*) (Nipponbare)	*OsPMM*	2466 ^e^	747 ^f^	+	248

^a ^The genomic DNA (gDNA) and cDNA sequences include start (ATG) and stop (TGA) codons.

^b ^Plant materials from which *PMM *sequences were isolated.

^c ^ORF disrupted by premature stop codon.

^d ^ORF interrupted by frameshift mutation.

^e ^The chromosomal locus containing *OsPMM *genomic sequence is *Os04g0682300*.

^f ^The GenBank accession number for *OsPMM *cDNA is DQ442996.

Six different *PMM *genes were isolated from each of the two bread wheat varieties, Xiaoyan 54 and Chinese Spring (CS). *T. urartu*, *Ae. tauschii *and barley were each found to contain two distinct *PMM *genes, while four different *PMM *genes were cloned from *T. turgidum*. Based on the data on exon and intron pattern, nucleotide and deduced protein identities, chromosomal location, and phylogenetic clustering analysis of genomic coding sequences (see below), the A, B and D genomes were each found to contribute two members to the six *PMM *genes in bread wheat, which were thus named as *TaPMM-A1*, *B1*, *D1*, *A2*, *B2 *and *D2*, respectively (Table 1). The *PMM-1 *genes in bread wheat and its diploid and tetraploid progenitor species and barley were homoeologous. Likewisely, homoeologous relationship existed among the *PMM-2 *genes from these species.

**Figure 1:**
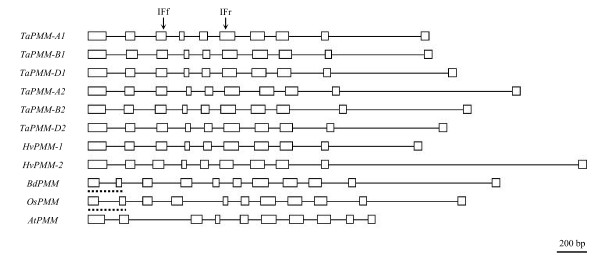
**Comparisons of the exon (box) and intron (line) patterns of the *PMM *genes from bread wheat (*TaPMM-A1*, *B1*, *D1*, *A2*, *B2 *and *D2*), barley (*HvPMM-1 *and *2*), *B. distachyon *(*BdPMM*), rice (*OsPMM*), and *Arabidopsis *(*AtPMM*)**. The exon/intron structures of the compared *PMM *genes are highly similar except that the first exon in *BdPMM *and *OsPMM *(underlined with dashed line) is split into two parts. The positions to which the intron flanking primers (IFf, IFr) bind are indicated by arrows. The chromosomal loci containing the genomic coding sequences of *AtPMM *and *OsPMM *are *At2g45790 *and *Os04g0682300*, respectively.

The intron and exon patterns of the *PMM *genes from rice, *B. distachyon*, and wheat and relatives were highly similar. Except for *OsPMM *and *BdPMM*, both of which had 11 exons owing to the split of the first exon into two parts, the remaining genes all had 10 exons and 9 introns (Figure 1). Apart from *PMM-A2 *homoeologs and *HvPMM-2*, the remaining *Triticeae PMM *genes cloned in this work all contained intact ORF. The coding region of *HvPMM-2 *was disrupted by premature stop codon based on analyzing the PCR products amplified from two barley varieties (Betzes, Zaoshou 3) (Table 1, Additional file 1).

Consistent with our earlier study [6], the proteins deduced from the *PMM *genes of common wheat and progenitor species, barley and *B. distachyon *were more than 50% identical to the PMMs of human and *S. cerevisiae *(Additional file 2). The identities among the grass PMMs were generally above 90%, with BdPMM showing higher identity to its homologs from bread wheat and related *Triticeae *species than to OsPMM. The identities among the deduced PMM-1 proteins of bread wheat and its diploid and tetraploid ancestors were generally 98% or higher, with complete identity found among the deduced TaPMM-A1, TuPMM-A1, TtPMM-A1 and TaPMM-B1 proteins. The identities among the deduced PMM-B2 and D2 proteins of bread wheat and its diploid and tetraploid ancestors were generally 97%. By contrast, the identities between the deduced PMM-1 and 2 proteins were comparatively lower, being 95% between B1 and B2 proteins, and 96% between D1 and D2 proteins. The deduced PMM-B1 (or B2) proteins of tetraploid and bread wheats were 99% identical. Interestingly, TaPMM-B2 and TtPMM-B2 displayed unusual amino acid substitutions. The first one, involving the substitution of a broadly conserved glycine residue by alanine, was shared between TaPMM-B2 and TtPMM-B2 (Additional file 3). The second one was unique to TaPMM-B2, involving the replacement of a highly conserved arginine residue by cysteine. The four structural and active site motifs (motifs I to IV), identified by analyzing the crystal structure of HsPMM-1 [7], were generally conserved among the compared plant PMMs (Additional file 3).

### Determination of *PMM *copy number and chromosomal locations

To investigate the copy number of *PMM *genes, we made use of intron length polymorphisms and designed a pair of intron flanking (IF) primers (Additional file 4), which enabled the amplification of PCR fragments containing introns III to V (Figure 1). By carefully analyzing the products amplified with fluorescently labeled IF primers, six distinct fragments were found for bread wheat (varieties Xiaoyan 54 and CS), and their size equaled to that calculated from the six cloned *TaPMM *genomic sequences in all cases (Additional file 5). Using the same strategy, two *PMM *fragments were found for *T. urartu *(accessions DV877 and IE29-1), *Ae. tauschii *(accessions AS67 and AS91) and barley (Betzes), respectively, and for all three species, the size of the amplified fragments corresponded exactly to the one deduced from the cloned *PMM *members (Additional file 5). Four fragments were amplified from *T. turgidum *(Langdon, LDN), whose size again showed specific correspondence to that derived from each of the four cloned *TtPMM *members (Additional file 5). Finally, a single fragment was amplified from *B. distachyon *(Bd21) and its size agreed with the one calculated from the cloned *BdPMM *genomic sequence (Additional file 5).

The copy number of *PMM *genes in an additional set of materials (containing 12 diploid and 9 tetraploid *Triticeae *species) was also investigated by fragment analysis using the IF primers (see above). The results showed that the diploid *Triticeae *species carrying the A^m^A^m^, CC, MM, NN, RR, SS, S^b^S^b^, S^l^S^l^, S^s^S^s^, S^sh^S^sh^, S^t^S^t ^or UU genomes generally had two different *PMM *fragments, whereas the tetraploid *Triticeae *species containing the AAGG, DDCC, DDMM, DDNN or UUMM genomes generally possessed four *PMM *specific fragments (Additional file 6).

The fluorescently labeled IF primers were further employed for investigating the chromosomal locations of wheat and barley *PMM *members by PCR amplifications. The fragments derived from *PMM-A1*, *B1 *and *D1 *of bread wheat were not amplified from the NT lines lacking 2A, 2B and 2D chromosomes, respectively (Additional file 7), indicating that *TaPMM-1 *homoeologs were located on group 2 chromosomes. This finding was confirmed by the fragment analysis data obtained with LDN substitution lines (Additional file 7). The fragments derived from *PMM-A2 *and *D2 *members of bread wheat were not amplified from the NT lines lacking 4A and 4D chromosomes, respectively (Additional file 7). Moreover, the fragment from *TtPMM-B2 *was absent in the LDN substitution line lacking the 4B chromosome (Additional file 7). Together, these results indicated that *TaPMM-2 *homoeologs were located on group 4 chromosomes. Using a similar strategy, the two barley *PMM *genes were mapped to the 2H and 4H chromosomes, respectively (Additional file 8), which are homoeologous to wheat groups 2 and 4 chromosomes, respectively [27,28].

The finding of six *PMM *genes located on groups 2 and 4 chromosomes in bread wheat by this work is consistent with the data generated in the wheat expressed sequence tag (EST) mapping project http://wheat.pw.usda.gov/NSF/progress_ mapping.html. The EST BE489906, a 421 bp cDNA exhibiting higher than 95% nucleotide sequence identity to the 5' half of the mRNAs of the six bread wheat *PMM *genes, has been used as a probe to hybridize with the genomic DNA samples of CS and associated NT lines digested with the restriction enzyme *Eco*RI. The hybridization yields 11 positive bands, which are mapped to groups 2 or 4 chromosomes. Our bioinformatic analysis suggests the presence of three to five *Eco*RI sites in the genomic ORF of bread wheat *PMM *genes, with the last *Eco*RI site located in the 8th exon (Additional file 9). Because the first seven exons give rise to the 5' half of *PMM *mRNAs upon post transcriptional processing, the DNA blot hybridization with BE489906 probe is expected to show about 11 to 12 hybridizing bands from six *TaPMM *gene members. The hybridization with another *PMM *EST probe (BE591860) yields approximately seven positive bands, which are mapped mainly to groups 2 or 4 chromosomes. Consequently, it is suggested that the number of homoeologous *PMM *genes in bread wheat is likely to be around six. However, we can not exclude the existence of additional and divergent *PMM *copies or fragments in bread wheat genome, because they may not be amplified by our PCR strategy (owing to mutations in the primer binding sites), or be distinguishable from one or more of the six completely sequenced copies during fragment analysis (because of the lack of difference in intron length).

### Duplication of *PMM *in diploid *Triticeae *species

**Figure 2:**
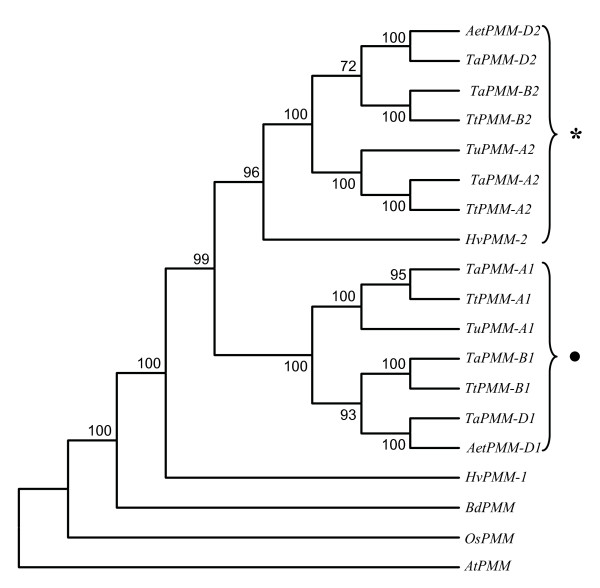
**Phylogenetic analysis of *PMM *genes**. The *PMM *sequences from bread wheat (*TaPMM-A1*, *B1*, *D1*, *A2*, *B2*, and *D2*), *T. turgidum *(*TtPMM-A1*, *B1*, *A2*, and *B2*), *T. urartu *(*TuPMM-A1 *and *A2*), *Ae. tauschii *(*AetPMM-D1 *and *D2*), barley (*HvPMM-1 *and *2*), *B. distachyon *(*BdPMM*), rice (*OsPMM*, chromosomal locus *Os04g0682300*), and *Arabidopsis *(*AtPMM*, chromosomal locus *At2g45790*) were used for this analysis. The filled dot and asterisk indicate the two clades composed of *Triticeae PMM-1 *and *2 *homoeologs, respectively. The tree shown was constructed based on an alignment of the nucleotide sequences of *PMM *genes by neighbor joining method (with P distance and complete deletion options). Highly similar results were obtained by alternative tree building programs (UPGMA, minimum evolution) (data not shown). The bootstrap values were each estimated using 500 replications. *AtPMM *was used as an outgroup control.

The foregoing experiments indicated that there existed two *PMM *genes (*PMM-1*, *PMM-2*) in diploid *Triticeae *species, and a single *PMM *gene in either *B. distachyon *or rice. These findings raised an important question on how and when the two *PMM *gene members in diploid *Triticeae *species were evolved. To investigate this question, phylogenetic analysis was conducted using the genomic DNA sequences of *PMM *genes. Two distinct clades, one composed of *Triticeae PMM-2 *homoeologs and the other of the *PMM-1 *homoeologs from common wheat and progenitors, were consistently found in the phylogenetic trees constructed using different tree building methods (Figure 2). *HvPMM-1*, *BdPMM *and *OsPMM *fell outside of the two clades, with *HvPMM-1 *showing a closer association with both clades than *BdPMM *and *OsPMM *(Figure 2). These observations, plus the established synteny between barley chromosome 2 (harboring *HvPMM-1*) and rice chromosome 4 (on which *OsPMM *resides) [27-29], indicated that the *Triticeae PMM-1 *genes were most likely the orthologs of *BdPMM *and *OsPMM*, and that the *Triticeae PMM-2 *member may be derived from *PMM-1 *through duplication. Trees with topology highly similar to that displayed in Figure 2 were also obtained when the deduced amino acid sequences of the *PMM *genes were used for phylogenetic analysis, but they could not reveal the phylogenetic relationships of *PMM-A2 *homoeologs to other *PMM *genes because *TaPMM-A2, TtPMM-A2 *and *TuPMM-A2 *could not yield intact proteins upon conceptual translation (Table 1).

To find the approximate timing at which the duplication of *Triticeae PMM *occurred, we first calculated the number of substitutions per synonymous site for the grass *PMM *genes characterized in this work. The values of synonymous substitutions calculated with *PMM-1 *genes were generally lower compared to those obtained with *PMM-2 *members (Table 2). This prompted us to calculate the molecular clocks for grass *PMM-1 *and *2 *genes, respectively. Based on the averaged synonymous substitutions between *OsPMM *and wheat *PMM-1 *or *2 *genes (Table 2), and assuming that rice and wheat diverged about 60 MYA [22], the average nucleotide substitution rate for *PMM-1 *genes was estimated to be 3.7 × 10^-9 ^per site per year, whereas that for *PMM-2 *genes was 4.2 × 10^-9 ^per site per year. *BdPMM *and wheat *PMM-1 *genes diverged about 40.2 ± 6.6 MYA (Table 3). The divergence time between wheat *PMM-1 *and *2 *genes was estimated to be 18.9 ± 4.0 or 16.6 ± 3.6 MYA, depending on whether the clock rates of *PMM-1 *or *2 *genes were used for the calculation (Table 3).

**Table 2:** Relative synonymous substitutions of *PMM *genes from rice, *B. distachyon*, bread wheat, and barley ^a^

*PMM *genes compared	Number of substitutions per synonymous site (dS)	Averaged dS
*OsPMM*/*BdPMM*	0.4231 ± 0.0646	
*OsPMM*/*HvPMM-1*	0.4807 ± 0.0723	
*OsPMM*/*TaPMM-A1*	0.4324 ± 0.0656	0.4385 ± 0.0669 (*OsPMM*/*TaPMM1 *genes)
*OsPMM*/*TaPMM-B1*	0.4573 ± 0.0701	
*OsPMM*/*TaPMM-D1*	0.4259 ± 0.0651	
*OsPMM*/*TaPMM-B2*	0.5150 ± 0.0791	0.4997 ± 0.0763 (*OsPMM*/*TaPMM2 *genes)
*OsPMM*/*TaPMM-D2*	0.4844 ± 0.0735	
*BdPMM*/*HvPMM-1*	0.3449 ± 0.0542	
*BdPMM*/*TaPMM-A1*	0.2884 ± 0.0471	0.2974 ± 0.0486 (*BdPMM*/*TaPMM1 *genes)
*BdPMM*/*TaPMM-B1*	0.3121 ± 0.0508	
*BdPMM*/*TaPMM-D1*	0.2918 ± 0.0479	
*BdPMM*/*TaPMM-B2*	0.3647 ± 0.0583	0.3503 ± 0.0562 (*BdPMM*/*TaPMM2 *genes)
*BdPMM*/*TaPMM-D2*	0.3358 ± 0.0541	
*HvPMM-1/TaPMM-A1*	0.0921 ± 0.0229	0.0971 ± 0.0237 (*HvPMM-1*/*TaPMM1 *genes)
*HvPMM-1/TaPMM-B1*	0.1126 ± 0.0261	
*HvPMM-1/TaPMM-D1*	0.0867 ± 0.0222	
*HvPMM-1/TaPMM-B2*	0.1857 ± 0.0355	0.1826 ± 0.0351 (*HvPMM-1*/*TaPMM2 *genes)
*HvPMM-1/TaPMM-D2*	0.1795 ± 0.0346	
*TaPMM-B1*/*TaPMM-B2*	0.1567 ± 0.0323	0.1398 ± 0.0299 (*TaPMM1*/*TaPMM2 *genes)

^a ^The dS values and their standard deviations were calculated using *PMM *cDNA coding sequences. *TaPMM-A2 *was not included in the calculation owing to the occurrence of mutation in its coding region.

**Table 3:** Divergence time estimates for *PMM *genes

	Divergence time (MYA)	Averaged dS value used for the estimation
*BdPMM *vs *TaPMM-1*	40.2 ± 6.6 ^a^	0.2974 ± 0.0486 (*BdPMM*/*TaPMM-1 *genes)
*TaPMM-1 *vs *TaPMM-2*	18.9 ± 4.0 ^a^	0.1398 ± 0.0299 (*TaPMM-1*/*TaPMM-2 *genes)
	16.6 ± 3.6 ^b^	

^a ^Specific molecular clock used for the estimation: 3.7 × 10^-9 ^per site per year.

^b ^Specific molecular clock: 4.2 × 10^-9 ^per site per year.

### Analysis of evolutionary rate and potential positive selection

The cDNA sequence of 11 *PMM *genes (including seven *PMM-1 *and four *PMM-2 *members from bread wheat and its progenitor species) were aligned (Additional file 10), and the resultant alignment was used to construct a NJ tree (Additional file 10). The non-synonymous/synonymous substitution rate ratios (*ω *= *d*_N_/*d*_S_) were calculated for analyzing evolution rate and potential positive selection on amino acid sites by appropriate models (Table 4). The analysis with one-ratio model (M0) produced an averaged *ω *value of 0.15, indicating that purifying selection dominated the evolution of the examined *PMM *gene members (Table 4). The analysis with two-ratio model revealed that the mean *ω *value of *PMM-2 *genes was larger than that of *PMM-1 *members, though this difference was not statistically significant by the likelihood-ratio test (LRT) comparing one-ratio with two-ratio models (Table 4). The analysis with six site specific models, M1a, M2a, M3 (*k *= 2), M3 (*k *= 3), M7 and M8, suggested that more than 90% of codon sites were under strong purifying selection (with *p *ranging from 0.91 to 0.99, Table 4). Two candidate sites (10 and 96) were found to be under positive selection based on naïve empirical Bayes (NEB) posterior probability, one of which (site 10) was significant (Table 4, Additional file 10). However, the positive selection on site 10 was not significant according to Bayes empirical Bayes (BEB) posterior probability, and neither was it supported by LRT (Table 4).

**Table 4:** Maximum-likelihood analysis of *PMM-1 *and *2 *genes and detection of codon sites under positive selection

						Likelihood ratio test (LRT)		
						
Model	*P*	Likelihood	Parameter estimate ^a^	NEB ^b^	BEB ^b^	2Δ*l*	df	*P *value
M0 (one ratio)	1	-1433.52	*ω *= 0.155	NA	NA			
Two ratio	2	-1433.09	*ω*_PMM-1 _= 0.114; *ω*_PMM-2 _= 0.186	NA	NA	0.4223 (two ratio versus one ratio)	1	0.5158
M1a: nearly neutral	1	-1430.84	*p*_0 _= 0.917; *p*_1 _= 0.083; *ω*_0 _= 0.076; *ω*_1 _= 1.000	NA	NA			
M2a: positive selection	3	-1429.69	*p*_0 _= 0.989; *p*_1 _= 0.000; *p*_2 _= 0.011; *ω*_0 _= 0.119; *ω*_1 _= 1.000; *ω*_2 _= 4.902	10N (0.999*); 96 H (0.793)	10N (0.867); 96 H (0.680)	2.2947 (M2a versus M1a)	2	0.3175
M3: discrete (*k *= 2)	3	-1429.69	*p*_0 _= 0.989; *p*_1 _= 0.011; *ω*_0 _= 0.119; *ω*_1 _= 4.901	10N (0.999*); 96 H (0.793)	None			
M3: discrete (*k *= 3)	5	-1429.69	*p*_0 _= 0.933; *p*_1 _= 0.056; *p*_2 _= 0.011; *ω*_0 _= 0.119; *ω*_1 _= 0.120; *ω*_2 _= 4.902	10N (0.999*); 96 H (0.793)	None	7.6562 (M3 versus M0)	4	0.1050
M7: beta	2	-1431.48	*p *= 0.0489; *q *= 0.241	NA	NA	NA		
M8: beta and *ω*	4	-1429.70	*p*_0 _= 0.989; *p *= 13.583; *q *= 99.000; *p*_1_= 0.011; *ω *= 4.909	10N (0.999*); 96 H (0.786)	10N (0.944); 96 H (0.778)	3.5539 (M7 versus M8)	2	0.1692

^a ^ω values are *d*_N_/*d*_S _ratios. *p*_0_, *p*_1 _and *p*_2 _indicate the proportion of sites in categories 1, 2 or 3.

^b ^The codon sites (10N, 96H) were numbered based on *TaPMM-D1 *cDNA (Additional file 10). Asterisk denotes statistical significance level at or above 0.95. NEB, naïve empirical bayes; BEB, Bayes empirical bayes; NA, not applicable.

### Transcriptional patterns of six *TaPMM *genes

**Figure 3:**
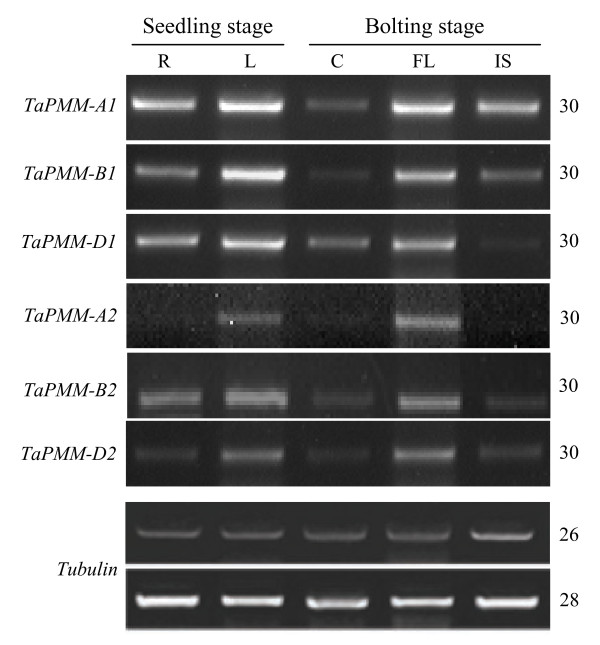
**Comparisons of the transcript levels of six *TaPMM *genes (*A1*, *B1*, *D1*, *A2*, *B2*, and *D2*) in Xiaoyan 54 organs collected at the seedling and bolting stages**. The total RNA samples, extracted from the roots (R), seedling leaves (L), culms (C), flag leaves (FL), and immature spikes (IS), were converted into cDNAs through reverse transcription, followed by semiquantitative RT-PCR analysis with gene specific primers. The amplification of *tubulin *transcripts served as an internal control (for normalizing the cDNA levels of different reverse transcription samples and for checking the kinetics of PCR amplification). The numbers of amplification cycles for *TaPMM *and *tubulin *genes are shown on the right side of the graph. The data depicted are representative of three independent experiments.

Semiquantitative RT-PCR experiments, using member specific primers, were conducted to compare the transcriptional patterns of six *PMM *genes in Xiaoyan 54. In general, the transcripts of the six *PMM *members were detectable in both vegetative (root, culm, leaf) and reproductive (immature spikes) organs, with relatively higher transcript levels detected in the seedling and flag leaves (Figure 3). The transcript levels of *TaPMM-1 *members (*A1*, *B1*, *D1*) were generally and relatively higher than those of *TaPMM-2 *members (*A2*, *B2*, *D2*) (Figure 3). The transcriptional profiles of the three *TaPMM-1 *genes were basically similar, except that the transcript level of *D1 *in the immature spikes was much lower relative to those of *A1 *or *B1 *(Figure 3). Among the three *TaPMM-2 *members, *B2 *and *D2 *showed a highly similar transcriptional profile (Figure 3). The transcript levels of *A2 *in the roots, culms and immature spikes were substantially lower than those of *B2 *and *D2 *in the same set of organs (Figure 3).

### Biochemical activity of TaPMM proteins

**Figure 4:**
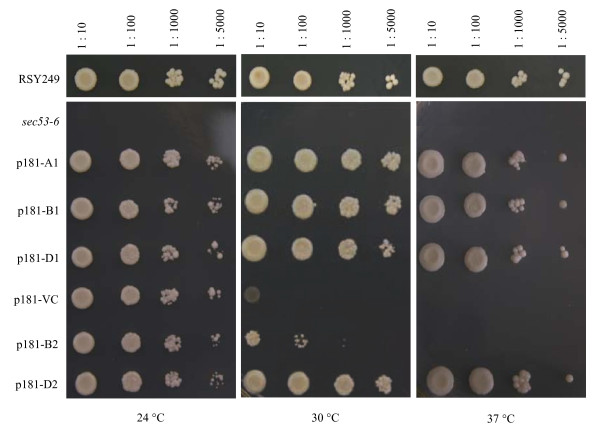
**Yeast complementation analysis of *TaPMM *genes**. Five yeast constructs, harboring the cDNA of *TaPMM-A1 *(p181-A1), *B1 *(p181-B1), *D1 *(p181-D1), *B2 *(p181-B2) or *D2 *(p181-D2), were prepared using the expression vector p181AINE (containing a *Leu2 *selection marker). These constructs, together with the empty vector control (p181-VC), were transformed into the *sec53-6 *strain of *S. cerevisiae*, which does not grow at elevated temperatures (30°C or above) because of temperature sensitive mutation in the native PMM. The six recombinant strains, *sec53-6*, and RSY249 (the wild type progenitor of *sec53-6*) were serially diluted and spotted onto the medium lacking leucine, followed by incubation at three test temperatures (24, 30 or 37°C), The data shown are reproducible in three independent experiments.

First, yeast complementation assays were carried out to test if the proteins expressed from *TaPMM-A1*, *B1*, *D1*, *B2 *or *D2 *may complement the temperature sensitivity of the *sec53-6 *mutant of *S. cerevisiae*. *TaPMM-A2 *was not included because it did not possess an intact ORF (Table 1). From Figure 4, it is clear that the expression of *TaPMM-A1*, *B1*, *D1 *or *D2 *restored the growth of *sec53-6 *cells at 30 or 37°C, and to a similar degree. By contrast, the expression of *TaPMM-B2 *complemented PMM deficiency of *sec53-6 *only weakly at 30°C, with no complementation observed at 37°C (Figure 4).

Subsequently, *TaPMM-A1*, *B1*, *B2*, *D1 *and *D2 *were individually expressed in the bacterial cells, and the resultant recombinant proteins were purified using nickel affinity chromatography (Additional file 11). The recombinant proteins of A1, B2, D1 and D2 were examined for catalytic activity using mannose-1-phosphate as the substrate. The B1 recombinant protein was not included in this analysis because its deduced amino acid sequence was identical to that of A1 (Additional file 2). In three independent experiments, B2 did not show detectable catalytic activity. By contrast, the recombinant A1, D1 and D2 proteins were all catalytically active. The average *K*_m _(mM, mannose-1-phosphate) values calculated for recombinant A1, D1 and D2 proteins were 0.46 ± 0.02, 0.40 ± 0.02 and 0.48 ± 0.02, respectively.

### Temperature-activity profiles of the recombinant PMMs of rice, *B. distachyon*, barley and bread wheat

**Figure 5:**
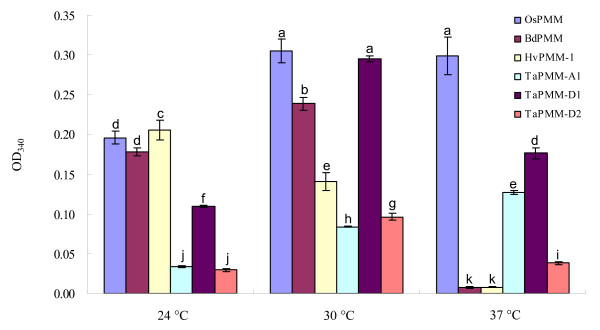
**Temperature-activity characteristics of the recombinant PMMs of bread wheat (TaPMM-A1, D1, and D2), barley (HvPMM-1), *B. distachyon *(BdPMM), and rice (OsPMM)**. The purified recombinant PMMs were each tested under three temperature conditions (24, 30 and 37°C), with mannose-1-phosphate as the substrate. The OD340 readings, recorded at 10 min after the start of the reaction, were used to compare the relative activity levels of the six PMMs under different temperatures. Means ± s.d. were each calculated using the results of triplicate measurements. Different letters above the bars suggest that the means differ significantly (P £ 0.05). The data shown are representative of three independent experiments.

*OsPMM*, *BdPMM *and *HvPMM-1 *were expressed in the bacterial cells, and the resultant recombinant proteins were purified as described above. The main temperature-activity characteristics of the three PMMs and TaPMM-A1, D1 and D2 were compared using 1 mM mannose-1-phosphate as the substrate and at three test temperatures (24, 30 and 37°C). From Figure 5, it is evident that the activity level of OsPMM was significantly higher at 30 and 37°C than at 24°C. Moreover, increasing the test temperature from 30 and 37°C did not decrease OsPMM activity significantly (Figure 5). BdPMM was most active at 30°C, but its activity level was reduced by nearly 95% as the temperature increased from 30 to 37°C (Figure 5). HvPMM-1 was most active at 24°C, and its activity level also decreased sharply (by more than 90%) as the temperature shifted from 30 to 37°C (Figure 5). The three bread wheat PMMs exhibited different temperature-activity characteristics. TaPMM-A1 was less active than BdPMM and HvPMM-1 at 24 and 30°C, but its activity level was substantially higher than those of BdPMM and HvPMM-1 at 37°C, owing to a 51% increase of its activity as the temperature changed from 30 to 37°C (Figure 5). TaPMM-D2, although less active than BdPMM and HvPMM-1 at 24 and 30°C, displayed substantially higher activity levels than BdPMM and HvPMM-1 at 37°C, because the change of test temperature from 30 to 37°C did not reduce TaPMM-D2 activity as strongly as that occurred to BdPMM and HvPMM-1 (Figure 5). The activity level of TaPMM-D1 was lower than those of BdPMM and HvPMM-1 but higher than those of TaPMM-A1 and D2 at 24°C (Figure 5). However, TaPMM-D1 was highly active at 30°C, with an activity level statistically comparable to that of OsPMM (Figure 5). The shift of assay temperature from 30 to 37°C reduced the activity of TaPMM-D1 by about 40%, but the scale of this reduction was much lower compared to that observed for BdPMM, HvPMM-1 or TaPMM-D2.

## Discussion

### *PMM *genes and chromosomal loci in *Triticeae *species

Through the molecular cloning experiments in this work, the following suggestions may be made regarding *PMM *genes and chromosome loci in *Triticeae *species. First, there exist two distinct *PMM *gene members (*PMM-1 *and *2*) in the progenitor diploid genomes (i.e., A, B and D) of bread wheat. There are four and six *PMM *genes (including both active and inactive members) in tetraploid and hexaploid wheats, respectively. Second, in bread wheat and its diploid and tetraploid progenitors, the loci containing *PMM-1 *or *2 *genes are located on homoeologous groups 2 and 4 chromosomes, respectively. Third, two *PMM *genes (*HvPMM-1 *and *2*) exist in diploid barley. The loci harboring *HvPMM-1 *or *2 *are located on the 2H and 4H chromosomes, respectively. Finally, judging from the fragment analysis data of *PMM *genes described in Additional file 6, it is probable that the basic organization of *PMM *gene family, as defined for the A, B, D and H genomes (e.g., two distinct members contained in two separate chromosomal loci), is conserved in the diploid *Triticeae *species possessing A^m^A^m^, CC, MM, NN, RR, SS, S^b^S^b^, S^l^S^l^, S^s^S^s^, S^sh^S^sh^, S^t^S^t ^or UU genomes. However, further work is needed to verify if in the 12 *Triticeae *genomes the two *PMM *genes may also be located on homoeologous group 2 and 4 chromosomes.

The existence of two *PMM *genes in diploid *Triticeae *species contrasts with the finding of a single *PMM *gene for *Arabidopsis*, rice and *B. distachyon *[[6,12], this work]. A search of recently accumulated genomic data deposited in the phytozome website (http://www.phytozome.net) reveals that *Medicago truncatula*, *Vitis vinifera*, *Sorghum bicolor *and *Zea mays *each possess a single *PMM *gene. However, both *Glycine max *and *Populus trichocarpa *harbor two *PMM *copies in their genomes (http://www.phytozome.net). Two functional *PMM *copies have also been found present in several mammals (e.g., human, mouse and rat) [30-32].

### Biochemically active PMM members in bread wheat and its progenitors

From the presence of intact ORF, the results of yeast complementation experiments, and the data of biochemical assays using recombinant proteins, we deduce that bread wheat expresses four biochemically active PMMs, TaPMM-A1, B1, D1 and D2. The primary structure and basic biochemical function of the four PMMs are similar to their orthologs in human, *S. cerevisiae *and *Arabidopsis*. In contrast to TaPMM-A1, B1, D1 and D2, TaPMM-B2 is probably not active *in vivo *because it did not complement PMM deficiency of yeast *sec53-6 *cells efficiently, and was not catalytically competent in the *in vitro *biochemical assay. The lack of detectable biochemical activity by TaPMM-B2 correlates with the presence of unusual amino acid substitutions in its deduced protein (Additional file 3). Further work is needed to determine the specific amino acid change(s) responsible for decreasing the biochemical activity of TaPMM-B2.

Owing to the possession of identical amino acid sequences (Additional file 2), it is likely that TuPMM-A1 (in *T. urartu*) behaves like TaPMM-A1, and AetPMM-D1 and D2 of *Ae. tauschii *may act like TaPMM-D1 and D2, respectively. Contrary to the above scenario, it is not possible to deduce the biochemical activity of TtPMM-B1 and B2 based on the data gathered for TaPMM-B1 and B2, because complete amino acid sequence identity did not exist between the two B1 or B2 members (Additional file 2).

### Main features of *PMM *evolution in diploid and polyploid *Triticeae *species

Despite its vital importance in eukaryotes, the molecular evolution of *PMM *has not been investigated in higher plants previously, and has been studied only recently in animals. Several investigations suggest that the presence of two functional *PMM *copies in human and murine cells may be caused by a gene duplication event that occurred 75 to 110 MYA [30,32-34]. From the data presented in this work, the main features in the evolution of *PMM *genes in diploid and polyploid *Triticeae *species may be summarized below. First, a major event in *PMM *evolution in the diploid *Triticeae *species carrying AA, A^m^A^m^, BB, CC, DD, HH, MM, NN, RR, SS, S^b^S^b^, S^l^S^l^, S^s^S^s^, S^sh^S^sh^, S^t^S^t ^or UU genomes is the duplication of *PMM*. This duplication event gave rise to two different *PMM *genes, *PMM-1 *and *2*, located on groups 2 and 4 chromosomes, respectively. Out of the two *PMM *genes in diploid *Triticeae *species, *PMM-1 *is likely more ancestral. This possibility is mainly supported by 1) the phylogenetic tree shown in Figure 2, 2) the existence of a single *PMM *gene in rice, and 3) the established syntenic relationship between *Triticeae *group 2 chromosomes (on which *PMM-1 *homoeologs are located) and rice chromosome 4 (on which *OsPMM *resides) [27-29]. Second, the *PMM *duplication, as discussed above, occurred after the divergence between *B. distachyon *and wheat, but before the radiation of diploid *Triticeae *genomes. This is supported by not only the presence of two different *PMM *gene members in many diploid *Triticeae *genomes examined in this work, but also the estimated divergence time between *TaPMM-1 *and *TaPMM-2 *genes (Table 3). The two estimates, whether 16.6 ± 3.6 or 18.9 ± 4.0 MY, are both much larger than the divergence time between wheat and barley (about 11 MY) reported in past studies [19-22]. But they are substantially smaller than the divergence time between *B. distachyon *and wheat (35 to 37.8 MY) calculated by previous investigations [35,36]. Third, substantial differences exist in the evolutionary patterns of *PMM-1 *and *2 *genes. *PMM-1 *genes generally had intact ORF, their transcript levels in the vegetative and reproductive organs were comparatively higher (Figure 3), and their protein products were all biochemically active (Figure 5). By contrast, multiple types of defects occurred to *PMM-2 *members. The *PMM-A2 *copies in bread wheat and its diploid and tetraploid progenitors and *HvPMM-2 *in barley possessed mutated ORF (Table 1). *TaPMM-B2 *encoded a protein with undetectable biochemical activity (Figure 4). The recombinant protein of *TaPMM-D2 *was significantly less active than that of the product of *TaPMM-D1 *(Figure 5). Moreover, the three *PMM-2 *members in bread wheat all exhibited lower transcript levels relative to their *PMM-1 *paralogs (Figure 3). Fourth, the differences of *PMM-1 *and *2 *genes established at the diploid level (i.e., in the A^u ^or D genomes) are basically retained in polyploid wheats. Thus, *PMM-A1 *is active in diploid, tetraploid and hexaploid levels whereas the ORF of *PMM-A2 *is disrupted at all three ploidy levels. The two *PMM *genes encoded in D genome are all active in both *Ae. tauschii *and bread wheat. In contrast to the *PMM *genes in A and D genomes, the origin of the contrasting biochemical features of the two B genome *PMM *members in bread wheat is unclear at present, because the absence of a diploid species containing the B genome made it difficult to study the evolution of *PMM-B1 *and *B2 *members at the diploid level. Finally, from the data described in Additional file 6, the duplicated *PMM *genes in the G, C, N, M and U genomes are also likely retained in the tetraploid species possessing AAGG, DDCC, DDMM, DDNN or UUMM genomes, although further work is needed to study the *PMM *genes in these species in more detail.

Because *PMM-1 *and *2 *genes were located on groups 2 and 4 chromosomes, respectively, in wheat and progenitors and barley, the duplication event that led to the formation of two *PMM *copies in the ancestral *Triticeae *species must have occurred interchromosomally. Strong evidence for the occurrence of interchromosomal duplications in wheat genome evolution has been obtained in recent years [37-39]. Importantly, it has been suggested that these duplications are largely conserved across different *Triticeae *species [39,40]. However, it is still not well understood how these events might be accomplished in *Triticeae *genomes. Thus, the *PMM *duplication reported here may serve as a useful model for studying the molecular mechanisms underlying the interchromosomal duplications in *Triticeae *in the future.

The average nucleotide substitution rates of grass *PMM-1 *or *2 *genes estimated based on the 60 MY divergence time between rice and wheat by this work are both lower than the commonly adopted mutation rate (6.5 × 10^-9 ^per site per year) for grass genes [41-44]. This may not be unusual because evolutionary rates frequently differ among different grass genes [45]. For example, the mutation rates of ten randomly selected grass genes have been found to vary from 4.1 to 7.1 × 10^-9 ^per site per year [45]. Furthermore, we estimated that the divergence time between *BdPMM *and wheat *PMM-1 *genes was about 40.2 ± 6.6 MY (Table 3) using the average nucleotide substitution rate calculated for grass *PMM-1 *genes. This estimate is comparable to the previously reported timeline (35 to 37.8 MY) for the divergence of the two species [35,36].

### Possible mechanisms involved in the evolution of *PMM-1 *and *2 *genes

Past studies have suggested several fates in the evolution of duplicated genes [46-48]. Neofunctionalization leads to the acquisition of new function by one copy, whereas subfunctionalization renders the progenitor and the duplicated copy acquiring non-overlapping but complementary expression patterns. Non-functionalization results in pseudogenization of one of the duplicates. In barley, the duplicate copy of *HvHox2 *(encoding a homeodomain-leucine zipper transcription factor) acquired a new function in controlling spikelet fertility [49]. In rice, evidence for subfunctionalization has been obtained for two C-class MADS box genes (*OSMADS3 *and *OSMADS58*) [50]. The evolutionary process of duplicated genes may be divided into two stages [51]. During the early stage, the duplicates generally evolve under purifying selection, with a phase of relaxed selective constraint immediately after the duplication [51,52]. During the later stage, the great majority of the duplicates become non-functional pseudogenes that are no longer expressed, with only a few of them being involved in neofunctionalization or subfunctionalization [51-53].

From the data in Table 4 and the main evolutionary features of *PMM-1 *and *2 *genes discussed above, we suggest that 1) purifying selection, but not positive selection, may have dominated the evolution of *PMM *gene family in wheat and relatives, and 2) despite the prevalence of purifying selection, the function of *PMM-2 *copies is not as strongly maintained as that of *PMM-1 *members. The first point is consistent with the findings that the vast majority of duplicated genes in cellular organisms experience purifying selection [51,52], even though some of them are in the process of becoming pseudogenes [54]. Owing to purifying selection, the *PMM-1 *genes are generally and relatively stable. By contrast, *TaPMM-A2 *is close to becoming a non-functional pseudogene, because its ORF was mutated and its transcript level was decreased relative to *PMM-1 *genes (Figure 3). This scenario may also apply to *PMM-A2 *copies in the diploid and tetraploid progenitors of bread wheat and *HvPMM-2 *in barley although further work is needed to check if the transcript levels of these members may be lowered than those of *PMM-1 *genes. *TaPMM-B2 *and *D2*, although possessing intact ORF, also show signs of degenerative evolution, because their transcript levels were lower than corresponding *PMM-1 *members, and the PMM enzyme activity of their recombinant proteins was either undetectable or reduced (Figure 4 and 5). The degeneration of *PMM-2 *genes and the comparatively high mean evolutionary rate of these members indicate the occurrence of relaxed purifying selection in the evolution of *PMM *gene family in wheat and relatives, which may have affected *PMM-2 *copies more strongly than *PMM-1 *paralogs. However, further work is needed to verify this hypothesis.

In contrast to the degenerative evolution of the duplicated *PMM-2 *copies in diploid *Triticeae *species, the two mammalian *PMM *members, products of a duplication event occurred before the radiation of mammals (i.e., 75 to 110 MYA), all encode highly active enzymes [29-33]. However, unlike *PMM2 *whose mutation leads to hypoglycosylation of cellular proteins and severe disease [9,12], the deletion of *PMM1 *in mouse does not cause obvious abnormalities in either growth or development under normal conditions [33]. Moreover, despite their overlapping expression profiles, *PMM1 *does not compensate for the functional loss of *PMM2 *[32,33]. These findings have led to the suggestion that *PMM1 *may have diverged from *PMM2 *in physiological function [33]. This possibility has been confirmed with the recent discovery that PMM1 but not PMM2 acts as a glucose-1,6-bisphosphatase in the brain tissues [55]. Clearly, the unique *PMM *duplication event in *Triticeae *plants revealed in this work differs from the one in mammals in multiple aspects including the timing of the event, the evolutionary fates of *PMM *members after the duplication, and possibly the main mechanism involved in the evolution. The evolutionary knowledge generated in this work increases our understanding of *PMM *evolution in eukaryotes, and may be useful for future studies of *PMM *evolution in additional plant species such as *G. max *and *P. trichocarpa*, both of which have been found to contain two *PMM *genes (see above).

### The main active *PMM *isozymes in bread wheat tissues

Among the four biochemically active TaPMMs, functional similarity may be limited to only TaPMM-A1 and B1 because their deduced proteins were identical, and the transcriptional patterns of their coding genes were very similar (Figure 3). By contrast, functional differences are likely to exist among A1(B1), D1 and D2 members because of dissimilarities in their deduced amino acid sequences and transcriptional patterns. For further analysis of the physiological function of *PMM *genes in bread wheat, it is necessary to find out the main active PMM isozymes *in vivo*. Considering the fundamental importance of PMM in the temperature adaptability of eukaryotes [1,12], the differences in the temperature-activity profiles of the four enzymatically active TaPMMs are of particular interest. From the data shown in Figure 5, it is suggested that TaPMM-D1 may be highly active in a wider range of temperatures (from 24 to 37°C). The activity of TaPMM-A1 is less than that of TaPMM-D1, but it is unique in exhibiting the highest activity at 37°C (although this activity level was still below that of TaPMM-D1 at 37°C). The activity of TaPMM-D2 is generally lower than that of TaPMM-D1 and A1, especially at 37°C (Figure 5). This finding and the observation that the transcript levels of *TaPMM-D2 *were considerably lower than those of *TaPMM-A1*, *B1 *and *D1 *in multiple organs (Figure 3) indicate that TaPMM-A1, B1 and D1 are likely to be the main active PMM isozymes in bread wheat tissues. Moreover, judging from Figure 3, at least two main PMM isozymes are expressed in each of the five organs (root, seedling leaf, culm, flag leaf, and developing spike) examined in this work.

As displayed in Figure 5, rice, a tropical cereal and adaptable to both tropical and temperate regions [56], possesses a PMM showing very high activity levels at elevated test temperatures (30 and 37°C). By contrast, *B. distachyon*, barley and bread wheat, originated from temperate regions [57-59], have the PMMs exhibiting relatively high activity levels at 24 or 30°C (Figure 5). A general decrease in the activities of the PMMs of *B. distachyon*, barley and bread wheat at 37°C seems to correlate with the temperate origin of these species. However, compared with BdPMM and HvPMM-1, at 37°C, the magnitude of activity decrease was much smaller for TaPMM-D1 and D2, and the activity of TaPMM-A1 even increased relative to its performance at 24 and 30°C. This indicates that bread wheat PMM isozymes may be generally more stable than their counterparts in *B. distachyon *and barley at elevated test temperatures (such as 37°C), which correlates well with the wide temperature adaptability of the bread wheat crop [13,14].

## Conclusion

This work has generated new information on *PMM *genes and their evolution in bread wheat and related *Triticeae *species. The main active PMM isozymes (e.g., TaPMM-A1, B1 and D1) in bread wheat tissues are revealed. The three isozymes may be more tolerant to elevated temperatures than their counterparts in barley and *B. distachyon*. The insights gained in this work have broadened the understanding of *PMM *evolution in eukaryotic organisms. The resources produced here may aid future investigations of the physiological function of this important gene in the temperature adaptability of bread wheat.

## Methods

### Plant materials, yeast strains and oligonucleotide primers

The wheat and barley varieties and the *T. urartu*, *T. turgidum *ssp. *dicoccoides*, *Ae. tauschii *and *B. distachyon *genotypes used for cloning *PMM *genes are listed in Additional file 1. The NT lines of CS, the D genome substitution lines in the tetraploid wheat variety LDN background, and the barley chromosome addition lines in CS background, employed for mapping the chromosomal locations of *PMM *genes, are also listed in Additional file 1. The growth of wheat and related *Triticeae *species was accomplished as described previously [6]. *B. distachyon *was cultured in the greenhouse under a constant temperature (23°C) and with a 16 h light/8 h dark photoperiod. Two *S. cerevisiae *strains, *sec53-6 *(*MAT a, sec53-6, leu2-3, -112, ura3-52*) and its wild type progenitor RSY249 used for the yeast complementation experiment, were described previously [6]. The oligonucleotide primers used in this work are all listed in Additional file 4. The general molecular methods for handling nucleic acid and protein samples, PCR amplification, DNA cloning, and protein expression in bacterial cells were adopted from Sambrook and Russell [60]. High fidelity Taq DNA polymerases, LA-Taq (TaKaRa, China) and Advantage 2 Polymerase Mix (Clontech, USA), were used in PCR amplifications. The accuracy of the yeast or bacterial expression constructs prepared in this work was all confirmed by DNA sequencing.

### Cloning of *PMM *cDNA and genomic DNA sequences

Total RNA samples were prepared from the desired leaf materials using RNeasy plant mini kit (Qiagen, Germany), and were converted into cDNAs using Moloney Murine leukemia virus (M-MLV) reverse transcriptase (Promega, USA). Public nucleotide and protein data banks were searched using the cDNA and deduced amino acid sequence of rice *PMM *(*OsPMM*) [6], leading to the finding of the ESTs derived from wheat and barley *PMM *genes. By analyzing the obtained ESTs, the oligonucleotide primers permitting the amplifications of full length cDNA and genomic DNA sequences of the *PMM *genes from bread wheat and related *Triticeae *species (*T. urartu*, *T. turgidum*, *Ae. tauschii*, barley) were developed (Additional file 4). With these primers and the reverse transcription products described above, the *PMM *cDNAs from bread wheat and relatives were amplified by RT-PCR, cloned and sequenced. The *PMM *cDNA of *B. distachyon *was cloned using a similar strategy, except that the development of the oligonucleotide primers specific for *BdPMM *was facilitated by the genome sequence of Bd21 (http://www.phytozome.net/brachy.php). Genomic DNA samples were extracted from the relevant leaf materials as detailed previously [61], and used for isolating the genomic coding sequences of the *PMM *genes of bread wheat and related *Triticeae *species and *B. distachyon *with the same sets of primers employed for cDNA cloning.

During the above experiments, at least 70 positive PCR clones derived from three separate cloning experiments were sequenced for each of the plant species examined in this work. This ensured the identification of the cDNA and genomic DNA sequences corresponding to different *PMM *genes. The final nucleotide sequences for the cDNA and genomic DNA coding regions of a given *PMM *gene were each constructed from the sequencing information of at least three independent clones. The nucleotide sequences of the *PMM *genes cloned in this work have all been submitted to GenBank. The accession numbers are listed in Additional file 12.

### Investigation of *PMM *copy number and chromosomal location

The genomic PCR products amplified with the IF primers (Additional file 4, Figure 1) from Xiaoyan 54 were confirmed to be derived from *PMM *genes by cloning and sequencing. The amplified fragments differed in size, which was thus useful for distinguishing the different *PMM *genes and estimating the copy number of *PMM *in bread wheat, its *Triticeae *relatives, and *B. distachyon*. The forward IF primer was labeled with D4 fluorescence by a commercial company (TaKaRa, China), allowing the separation and identification of PCR products via capillary electrophoresis (CE) and fragment analysis in the CEQ/GeXP Genetics Analysis System (GAS) (Beckman, USA). The preparation of PCR products for CE and the subsequent fragment analysis in CEQ/GeXP GAS were conducted following the supplier's instructions. Using the same strategy, *PMM *copy numbers in the *Triticeae *species carrying the diploid A^m^A^m^, CC, MM, NN, RR, SS, S^b^S^b^, S^l^S^l^, S^s^S^s^, S^sh^S^sh^, S^t^S^t ^or UU genomes, or the tetraploid AAGG, DDCC, DDMM, DDNN or UUMM genomes (Additional file 6), were also investigated.

For investigating *PMM *chromosomal location, the *PMM *gene specific fragments, amplified from CS, the different NT lines of CS, LDN, and the D genome substitution lines in LDN background, were separated using CE and differentiated by fragment analysis. The IF primers and fragment analysis were also used for mapping the chromosomal locations of two barley *PMM *genes (*HvPMM-1 *and *2*). In this case, the DNA samples extracted from the barley variety Betzes and its derivative chromosome addition lines in CS background were utilized as templates for genomic PCR amplifications.

### Bioinformatic, phylogenetic and evolutionary analysis of *PMM*

Bioinformatic investigations of cloned *PMM *genes were carried out using the softwares installed in public websites, including EBI (http://www.ebi.ac.uk/Tools/), ExPASy (http://www.expasy.ch/tools/) and NCBI (http://www.ncbi.nlm.nih.gov/). Full length genomic DNA sequences of *PMM *genes were aligned using the Clustalw2 program (EBI website). The resulted alignment was used for constructing phylogenetic trees by MEGA4 [62]. The number of substitutions per synonymous site (dS) was calculated as described previously [63]. The average nucleotide substitution rates for *PMM-1 *and *2 *genes were estimated by the formula k = dS/2t [64], assuming a divergence time of 60 MY between rice and wheat [22]. The obtained rates were then used to estimate the timing of *PMM *duplication in wheat.

To investigate the potential mechanisms behind the evolution of *PMM *genes in bread wheat and its progenitor *Triticeae *species, two complementary evolutionary tests (one for difference in the evolutionary rates of *PMM-1 *and *2 *genes, and the other for the codon sites under positive selection) were carried out by the CODEML program implemented in the PAML package [65]. The NJ tree, constructed using the cDNA sequence of 11 active *PMM-1 *and *2 *genes, was employed for these tests. The one-ratio and two-ratio models [66,67] were used for investigating evolutionary rate, whereas the site specific models M1a, M2a, M3 (*k *= 2), M3 (*k *= 3), M7 and M8 [68,69] were deployed for testing potential diversifying selection at individual codon sites. Statistical analysis of the evolutionary data was conducted as described previously, with the confidence level set at or above 0.95 [70,71].

### Semiquantitative RT-PCR

The seeds of Xiaoyan 54 were germinated for 48 h at 25°C, followed by 4 weeks vernalization treatment at 4°C. The vernalized seedlings were then grown in the greenhouse as described above. The root and seedling leaf samples were collected after two weeks growth in the greenhouse. The stem, flag leaf, and immature spike samples were collected at the bolting stage (after 5 weeks growth in the greenhouse). The isolation of total RNA samples from the collected materials and cDNA synthesis were conducted as described above. Semiquantitative RT-PCR analysis of the relative transcript levels of 6 *TaPMM *genes in different wheat organs was performed using gene specific primers (Additional file 4), and following the method detailed previously [72]. The amplification of wheat *tubulin *transcripts served as an internal control, and the reproducibility of the transcriptional patterns revealed was verified in three independent experiments.

### Yeast complementation

The complementation assay was conducted as described previously [6]. Briefly, five complementation constructs (p181-A1, B1, D1, B2 and D2) were prepared by cloning the cDNAs of *TaPMM-A1*, *B1*, *D1*, *B2 *and *D2 *individually into the yeast expression vector p181AINE. The oligonucleotide primers used for amplifying *TaPMM *cDNAs during the cloning are listed in Additional file 4. The five constructs, together with the empty vector control (p181-VC), were individually introduced into the *sec53-6 *strain whose endogenous *PMM *harbors a temperature sensitive mutation [1]. The recombinant strains were serially diluted and plated onto agar medium, followed by incubation at three temperatures (24, 30 and 37°C). As controls, *sec53-6 *and its wild type progenitor RSY249 were also included in the growth assays. Successful complementation by *TaPMM *expression was indicated by restoring the growth of *sec53-6 *cells at elevated temperatures (30 and 37°C). The complementation assay was repeated three times to verify the reproducibility of the data obtained.

### Biochemical analysis of recombinant PMMs

The *PMM *cDNAs from bread wheat (*TaPMM-A1*, *B1*, *D1*, *B2 *and *D2*), barley (*HvPMM-1*), *B. distachyon *(*BdPMM*) and rice (*OsPMM*) were individually cloned into the bacterial expression vector pET-30a (Novagen, USA), in order to express recombinant PMM proteins with a C-terminal histidine tag. The oligonucleotide primers used for amplifying *PMM *cDNAs during preparing the bacterial expression constructs are provided in Additional file 4. The induction of *PMM *cDNA expression in the bacterial cells and the purification of histidine tagged PMMs through metal chelate affinity chromatography were accomplished as described previously [6]. The purified PMMs were characterized using 10% SDS-PAGE, and were confirmed to be the desired histidine tagged proteins by protein blotting using an anti-histidine antibody (Roche Diagnostic GmbH, Germany) [6].

The kinetic properties of the purified recombinant TaPMM-A1, B2, D1 and D2 were investigated using mannose-1-phosphate as the substrate, and following the method detailed in our earlier work [6]. The assay was repeated three times for each of the four recombinant TaPMMs. The resultant data were used to calculate average *K*_m _values. The temperature-activity characteristics of OsPMM, BdPMM, HvPMM-1, and TaPMM-A1, D1 and D2 were examined using 1 mmol/L mannose-1-phosphate as the substrate, with the assays conducted at three different temperatures (24, 30 and 37°C). The OD_340 _readings were collected at 10 min after the start of the enzyme assays. At this time point, the reactions catalyzed by the six recombinant PMMs were in the linear phase. The assay was repeated three times per recombinant PMM per test temperature, with each assay consisting of three technical replicates. The OD_340 _readings obtained were statistically analyzed using the software SPSS 10 for comparing the relative activity levels of the six recombinant PMMs.

## Authors' contributions

CY participated in experimental design, cloned *PMM *genes, determined *PMM *copy number, and conducted RT-PCR, yeast complementation, protein purification and biochemical experiments. YL, BL, LH, JC, WQ, SL, GW, SB and HQ contributed resources, reagents and greenhouse facilities to experiments. XL and HY performed evolutionary analysis. QS, LC and AZ took part in designing and supervising the study. DW designed and supervised the study and participated in drafting the manuscript. All authors have read and approved the final manuscript.

## Supplementary Material

Additional file 1 **Plant materials**. The materials were used for *PMM *gene cloning and chromosomal localization experiments. Click here for file

Additional file 2 **Amino acid sequence identities**. The PMM proteins under comparison were from wheat and related species, yeast (*S. cerevisiae*, ScPMM), human (HsPMM1 and 2), *Arabidopsis *(AtPMM), rice (OsPMM) and *B. distachyon *(BdPMM). Click here for file

Additional file 3 **Alignment of the deduced amino acid sequences of the PMMs from wheat and related *Triticeae *species, *B. distachyon *(BdPMM), rice (OsPMM), and human (HsPMM1 and 2)**. The four structural motifs required for PMM catalysis are boxed. The replacement of a broadly conserved arginine residue by cysteine (marked in red) was found in TaPMM-B2, whereas the substitution of a highly conserved glycine residue by alanine (written in blue) was observed in both TaPMM-B2 and TtPMM-B2. Asterisks indicate identical residues. The symbols ":" and "." represent conserved and semi-conserved substitutions, respectively. The Swiss-Prot accession numbers for OsPMM, HsPMM1 and HsPMM2 are Q7XPW5, Q92871 and O15305, respectively. Click here for file

Additional file 4 **Oligonucleotide primers used in this work**. Click here for file

Additional file 5 **Investigation of *PMM *copy numbers in bread wheat and related *Triticeae *species (*T. urartu*, *Ae. tauschii*, *H. vulgare*, *T. turgidum *ssp. *durum*) and *B. distachyon *by fragment analysis**. *PMM *gene specific peaks are indicated by arrows. The minor peaks labeled by asterisks are caused by DNA size standards. The scale on the horizontal axis indicates fragment size (number of nucleotides), which was determined using the DNA size standards co-separated with the PCR products. The specific genotypes used in this analysis are provided in the brackets. The data shown are representative of five independent experiments. Click here for file

Additional file 6 **Estimation of *PMM *copy numbers in the diploid and tetraploid species possessing additional *Triticeae *genomes**. Click here for file

Additional file 7 **Chromosomal assignment of *PMM *genes in hexaploid and tetraploid wheats**. The fragments specific for individual *PMM *gene members were amplified by PCR, followed by separation via capillary electrophoresis. The templates for PCR were prepared from Chinese Spring (CS), the nulli-tetrasomic (NT) lines of CS, the durum wheat variety Langdon (LDN), and the D genome substitution lines in LDN background. The *PMM *genes represented by the major peaks (fragments) are shown. The minor peaks labeled by asterisks are due to DNA size markers. The scale on the horizontal axis indicates fragment size. (a) Compared to the presence of all six *PMM *specific fragments in CS, the fragments representing *PMM-A1*, *B1*, *D1*, *A2 *or *D2 *genes were specifically absent from the NT lines lacking chromosomes 2A (N2AT2D), 2B (N2BT2D), 2D (N2DT2A), 4A (N4AT4D) or 4D (N4DT4A). The six *PMM *specific fragments were all amplified in the NT lines lacking other groups (i.e., 1, 3, 5, 6 and 7) of chromosomes (data not shown). (b) The presence of four *PMM *specific fragments in LDN. Note that the fragments representing *PMM-A1*, *B1 *or *B2 *genes were not amplified from the substitution lines 2D(2A) (lacking 2A chromosome), 2D(2B) (lacking 2B chromosome) or 4D(4B) (lacking 4B chromosome). The fragment representing *PMM-D1 *was amplified from 2D(2A) and 2D(2B) owing to the presence of 2D chromosome in the two lines. Similarly, the fragment representing *PMM-D2 *was amplifiable from 4D(4B) due to the presence of 4D chromosome in this line. The *PMM-A1*, *B1*, *A2 *and *B2 *fragments were all amplifiable in the other substitution lines in which 2A, 2B, 4A and 4B chromosomes are not involved in the substitution by D genome chromosomes (data not shown). The data displayed are typical of five independent sets of experiments. Click here for file

Additional file 8 **Chromosomal locations of barley *PMM *genes (*HvPMM-1 *and *2*)**. The genomic DNA samples, extracted from the bread wheat variety Chinese Spring (CS), barley variety Betzes, and barley chromosome addition lines in CS background, were used for PCR amplifications with the nucleotide primers capable of recognizing *PMM *genes from *Triticeae *species. The peaks in blue represent PCR fragments amplified from specific bread wheat or barley *PMM *genes. The six *PMM *genes in CS are abbreviated as *A1*, *B1*, *D1*, *A2*, *B2 *and *D2*, respectively. *HvPMM-1 *and *2 *were found in the two addition lines containing barley 2H (CS-2H) and 4H (CS-4H) chromosomes, respectively. The fragment pattern of the remaining addition lines was identical to that of CS (data not shown). The peaks marked by asterisks were caused by DNA size standards. The data shown are typical of three independent experiments. Click here for file

Additional file 9 **Distribution of multiple *Eco*RI sites in the genomic ORFs of six bread wheat *PMM *genes**. The locations of the sites are calculated in relation to the start codon ATG (with the A nucleotide as 1). The exons are represented by boxes. Click here for file

Additional file 10 **The cDNA sequence alignment and phylogenetic genetic tree of *PMM *genes used for evolutionary rate and positive selection analysis**. (a) Multiple alignment of the cDNA sequences of 11 active *PMM *genes from bread wheat and its progenitor species. The sequences are each presented in codon format. The stop codon is not included in the alignment. Codon site 10 (numbered according to *TaPMM-D1*), which was significantly positively selected based on based on NEB posterior probability, is labeled in purple. (b) The phylogenetic tree used for evolutionary analysis. This tree was constructed using neighbor joining program with complete deletion and the Kimura-2 nucleotide substitution model. The bootstrap value was estimated using 500 replications. Click here for file

Additional file 11 **SDS-PAGE analysis of recombinant TaPMM-A1, B1, D1, B2, and D2 proteins purified using metal chelate affinity chromatography**. The size of protein molecular mass (kD) standards is shown on the left side of the graph. Click here for file

Additional file 12 **GenBank accession numbers of the *PMM *genes isolated in this work**. Click here for file
