# Supplementary material for: Molecular analysis of phosphomannomutase (PMM) genes reveals a unique PMM duplication event in diverse Triticeae species and the main PMM isozymes in bread wheat tissues
Source: BMC Plant Biol. 2010 Oct 5;10:214. doi: 10.1186/1471-2229-10-214 (PMC3017832; doi:10.1186/1471-2229-10-214)
Supplement: Additional file 1 — Plant materials. The materials were used for PMM gene cloning and chromosomal localization experiments. [file 1471-2229-10-214-S1.PDF]

Additional file 1: Plant materials used for *PMM* gene cloning and chromosomal localization experiments in this work

Species	Genome and ploidy level	Material
T. urartu	AA, 2n = 2x = 14	DV877, IE29-1
Ae. tauschii	DD, 2n = 2x =14	AS67, AS91
T. turgidum	AABB, 2n = 4x = 28	Langdon , the D genome substitution lines in Langdon background
T. aestivum	AABBDD, 2n = 6x = 42	Xiaoyan 54 , Chinese Spring , the nulli-tetrasomic (NT) lines of Chinese Spring
H. vulgare	HH, 2n = 2x = 14	Betzes, Betzes chromosome addition lines in Chinese Spring background, Zaoshou 3
B. distachyon	2n = 2x = 10	Bd21

The AS genotypes are from the *Triticeae* Research Institute, Sichuan Agricultural University, China. The DV and IE genotypes are from University of California-Davies, USA. The barley materials are supplied by Nanjing Agricultural University, China. Bd21 is from USDA Western Regional Research Center, Albany, USA.
