# Supplementary material for: Molecular analysis of phosphomannomutase (PMM) genes reveals a unique PMM duplication event in diverse Triticeae species and the main PMM isozymes in bread wheat tissues
Source: BMC Plant Biol. 2010 Oct 5;10:214. doi: 10.1186/1471-2229-10-214 (PMC3017832; doi:10.1186/1471-2229-10-214)
Supplement: Additional file 2 — Amino acid sequence identities. The PMM proteins under comparison were from wheat and related species, yeast (S. cerevisiae, ScPMM), human (HsPMM1 and 2), Arabidopsis (AtPMM), rice (OsPMM) and B. distachyon (BdPMM). [file 1471-2229-10-214-S2.PDF]

Additional file 2:

Amino acid sequence identities among the deduced PMM proteins from wheat and related species, yeast (*S. cerevisiae* , ScPMM), human (HsPMM1 and 2), *Arabidopsis* (AtPMM), rice (OsPMM) and *B. distachyon* (BdPMM)

	ScPMM	HsPMM1	HsPMM2	AtPMM	OsPMM	BdPMM	AetPMM-D2	TaPMM-D2	TaPMM-B2	TtPMM-B2	HvPMM-1	AetPMM-D1	TaPMM-D1	TaPMM-B1	TtPMM-B1	TtPMM-A1	TaPMM-A1	TuPMM-A1
ScPMM	100	56	58	58	55	56	54	54	54	55	54	54	54	55	55	54	54	54
HsPMM1		100	67	55	55	55	56	56	56	56	55	56	56	56	56	55	56	56
HsPMM2			100	60	59	59	58	58	57	58	59	59	59	58	58	58	59	59
AtPMM				100	81	80	79	79	79	79	80	79	79	79	79	79	79	79
OsPMM					100	91	90	90	90	89	89	90	90	90	90	90	90	90
BdPMM						100	94	94	93	93	93	94	94	94	94	94	94	94
AetPMM-D2							100	100	97	97	95	96	96	96	96	96	96	96
TaPMM-D2								100	97	97	95	96	96	96	96	96	96	96
TaPMM-B2									100	99	94	95	95	95	95	95	95	95
TtPMM-B2										100	94	94	94	94	95	94	95	95
HvPMM-1											100	98	98	98	98	98	98	98
AetPMM-D1												100	100	98	98	98	99	99
TaPMM-D1													100	98	98	98	99	99
TaPMM-B1														100	99	99	100	100
TtPMM-B1															100	98	99	99
TtPMM-A1																100	100	100
TaPMM-A1																	100	100
TuPMM-A1																		100

The Swiss-Prot accession numbers for ScPMM, HsPMM1, HsPMM2, AtPMM and OsPMM are P07283, Q92871, O15305, O80840 and Q7XPW5, respectively.
