# Supplementary material for: Molecular analysis of phosphomannomutase (PMM) genes reveals a unique PMM duplication event in diverse Triticeae species and the main PMM isozymes in bread wheat tissues
Source: BMC Plant Biol. 2010 Oct 5;10:214. doi: 10.1186/1471-2229-10-214 (PMC3017832; doi:10.1186/1471-2229-10-214)
Supplement: Additional file 3 — Alignment of the deduced amino acid sequences of the PMMs from wheat and related Triticeae species, B. distachyon (BdPMM), rice (OsPMM), and human (HsPMM1 and 2). The four structural motifs required for PMM catalysis are boxed. The replacement of a broadly conserved arginine residue by cysteine (marked in red) was found in TaPMM-B2, whereas the substitution of a highly conserved glycine residue by alanine (written in blue) was observed in both TaPMM-B2 and TtPMM-B2. Asterisks indicate identical residues. The symbols ":" and "." represent conserved and semi-conserved substitutions, respectively. The Swiss-Prot accession numbers for OsPMM, HsPMM1 and HsPMM2 are Q7XPW5, Q92871 and O15305, respectively. [file 1471-2229-10-214-S3.PDF]

Additional file 3

```
                              I                                  II
TtPMM-A1    MAAA----RKDAGVLALFDVDGTLTAPRKEVTPEMLEFMKRLRENVTVGVVGGSDLVKISEQLG--KSVITDYDYVFSENGLVAHKDGKLIGTQSLKTYLGDDQLKEFINFTLHYIAD
TuPMM-A1    MAAA----RKDAGVLALFDVDGTLTAPRKEVTPEMLEFMKRLRENVTVGVVGGSDLVKISEQLG--KSVITDYDYVFSENGLVAHKDGKLIGTQSLKTYLGDDQLKEFINFTLHYIAD
TaPMM-A1    MAAA----RKDAGVLALFDVDGTLTAPRKEVTPEMLEFMKRLRENVTVGVVGGSDLVKISEQLG--KSVITDYDYVFSENGLVAHKDGKLIGTQSLKTYLGDDQLKEFINFTLHYIAD
TaPMM-B1    MAAA----RKDAGVLALFDVDGTLTAARKEVTPEMLEFMKRLRENVTVGVVGGSDLVKISEQLG--KSVITDYDYVFSENGLVAHKDGKLIGTQSLKTYLGDDQLKEFINFTLHYIAD
TtPMM-B1    MAAA----RKNAGVLALFDVDGTLTAARKEVTPEMLEFMKRLRENVTVGVVGGSDLVKISEQLG--KSVITDYDYVFSENGLVAHKDGKLIGTQSLKTFLGDDQLKEFINFTLHYIAD
TaPMM-D1    MAAAAAA-RKTAGVLALFDVDGTLTAPRKEVTPEMLEFMKRLRENVTVGVVGGSDLVKISEQLG--KSVITDYDYVFSENGLVAHKDGKLIGTQSLKTYLGDDQLKEFINFTLHYIAD
AetPMM-D1   MAAAAAA-RKTAGVLALFDVDGTLTAPRKEVTPEMLEFMKRLRENVTVGVVGGSDLVKISEQLG--KSVITDYDYVFSENGLVAHKDGKLIGTQSLKTYLGDDQLKEFINFTLHYIAD
HvPMM-1     MAAAG----KSTGVLALFDVDGTLTAPRKEVTPEMLDFMKRLRENVTVGVVGGSDLVKISEQLG--KSVITDYDYVFSENGLVAHKDGKLIGTQSLKTYLGDDQLKEFINFTLHYIAD
TaPMM-B2    MAA-----KKNAGVLALFDVDGTLTAPCKEVTPELLEFMKRLRENVTVGVVGGSDLVKISEQLA--KSVITDYDYVFSENGLVAHKDGKLIGTQSLKTHLGDDQLKEFINFTLHYIAD
TtPMM-B2    MAA-----KKNAGVLALFDVDGTLTAPRKEVTPELLEFMKRLRENVTVGVVGGSDLVKISEQLA--KSVITDYDYVFSENGLVAHKDGKLIETQSLKTHLGDDQLKEFINFTLHYIAD
TaPMM-D2    MVASAA--RKNAGVLALFDVDGTLTAPRKEVTPEMLEFMKRLRENVTVGVVGGSDLVKISEQLG--KSVITDYDYVFSENGLVAHKDGKLIGTQSLKTYLGDDQLKEFINFTLHYIAD
AetPMM-D2   MVASAA--RKNAGVLALFDVDGTLTAPRKEVTPEMLEFMKRLRENVTVGVVGGSDLVKISEQLG--KSVITDYDYVFSENGLVAHKDGKLIGTQSLKTYLGDDQLKEFINFTLHYIAD
BdPMM       MAAV----KKNAGVLALFDVDGTLTAPRKEVTPEMLEFMRLLRENVTVGVVGGSDLVKISEQLG--KSVITDYDYCFSENGLVAHKDGKLIGTQSLKSYLGDDQLKDFINFTLHYIAD
OsPMM       MAA-----RKNAGVLALFDVDGTLTAPRKVVTPEMLQFMKQLREHVTVGVVGGSDLVKISEQLG--KSVTTDYDYCFSENGLVAHKNGELIGTQSLKSFLGDDQLKEFINFTLHYIAD
HsPMM1      MAVTAQAARRKERVLCLFDVDGTLTPARQKIDPEVAAFLQKLRSRVQIGVVGGSDYCKIAEQLGDGDEVIEKFDYVFAENGTVQYKHGRLLSKQTIQNHLGEELLQDLINFCLSYMAL
HsPMM2      MAAPGPA-------LCLFDVDGTLTAPRQKITKEMDDFLQKLRQKIKIGVVGGSDFEKVQEQLG--NDVVEKYDYVFPENGLVAYKDGKLLCRQNIQSHLGEALIQDLINYCLSYIAK
            *..           *.*********.. : :  *:  *:: **..: :*******  *: ***.  ..*  .:** *.*** * :*.*.*:  *.::..**:  ::::**: * *:*

                                                                                                  III                IV
TtPMM-A1    LDIPIKRGTFIEFRSGMINVSPIGRNCSQEERDDFEKYDKVHNVRPKMVSVLREKFTHLNLTFSIGGQISFDVFPQGWDKTYCLRYLEE--FKEIHFFGDKTYKGGNDHEIFESDRTV
TuPMM-A1    LDIPIKRGTFIEFRSGMINVSPIGRNCSQEERDDFEKYDKVHNVRPKMVSVLREKFAHLNLTFSIGGQISFDVFPQGWDKTYCLRYLEE--FKEIHFFGDKTYKGGNDHEIFESDRTV
TaPMM-A1    LDIPIKRGTFIEFRSGMINVSPIGRNCSQEERDDFEKYDKVHNVRPKMVSVLREKFAHLNLTFSIGGQISFDVFPQGWDKTYCLRYLEE--FKEIHFFGDKTYKGGNDHEIFESDRTV
TaPMM-B1    LDIPIKRGTFIEFRSGMINVSPIGRNCSQEERDDFEKYDKVHNVRPKMVSVLREKFAHLNLTFSIGGQISFDVFPQGWDKTYCLRYLEE--FKEIHFFGDKTYKGGNDHEIFESDRTV
TtPMM-B1    LDIPIKRGTFIEFRSGMINVSPIGRNCSQEERDDFEKYDKVHNVRPKMVSVLREKFAHLNLTFSIGGQISFDVFPQGWDKTYCLRYLEE--FKEIHFFGDKTYKGGNDHEIFESDRTV
TaPMM-D1    LDIPIKRGTFIEFRSGMINVSPIGRNCSQEERDDFEKYDKVHNVRPKMVSVLREKFAHLNLTFSIGGQISFDVFPQGWDKTYCLRYLEE--FKEIHFFGDKTYKGGNDHEIFESERTV
AetPMM-D1   LDIPIKRGTFIEFRSGMINVSPIGRNCSQEERDDFEKYDKVHNVRPKMVSVLREKFAHLNLTFSIGGQISFDVFPQGWDKTYCLRYLEE--FKEIHFFGDKTYKGGNDHEIFESERTV
HvPMM-1     LDIPIKRGTFIEFRSGMINVSPIGRNCSQEERDDFEKYDKVHNVRPKMVSVLREKFAHLNLTFSIGGQISFDVFPQGWDKTYCLRYLEE--FKEIHFFGDKTYKGGNDHEIFESDRTV
TaPMM-B2    LDIPIKRGTFIEFRSGMINVSPIGRNCSQEERDDFEKYDKVHNVRPKMVSVLREKFAHLNLTFSIGGQISFDVFPHGWDKTYCLRYLDE--FKEIHFFGDKTYKGGNDHEIFESDRTV
TtPMM-B2    LDIPIKRGTFIEFRSGMINVSPIGRNCSQEERDDFEKYDKVHNVRPKMVSVLREKFAHLNLTFSIGGQISFDVFPHGWDKTYCLRYLDE--FKEIHFFGDKTYKGGNDHEIFESDRTV
TaPMM-D2    LDIPIKRGTFIEFRSGMINVSPIGRNCSQEERDDFEKYDKVHNVRPKMVSMLREKFAHLNLTFSIGGQISFDVFPHGWDKTYCLRYLDE--FKEIHFFGDKTYKGGNDHEIFESDRTV
AetPMM-D2   LDIPIKRGTFIEFRSGMINVSPIGRNCSQEERDDFEKYDKVHNVRPKMVSMLREKFAHLNLTFSIGGQISFDVFPHGWDKTYCLRYLDE--FKEIHFFGDKTYKGGNDHEIFESDRTV
BdPMM       LDIPIKRGTFIEFRSGMINVSPIGRNCSQEERDDFEKYDKVHNIRPKMVSILREKFAHLNLTFSIGGQISFDVFPQGWDKTYCLRYLEE--FKEIHFFGDKTYKGGNDHEIFESSRTV
OsPMM       LDIPIKRGTFIEFRSGMLNVSPIGRNCSQEERDEFEKYDKVHNIRPKMVSVLREKFAHLNLTFSIGGQISFDVFPQGWDKTYCLRYLEE--FQEIHFFGDKTYKGGNDYEIFESDRTI
HsPMM1      LRLPKKRGTFIEFRNGMLNISPIGRSCTLEERIEFSELDKKEKIREKFVEALKTEFAGKGLRFSRGGMISFDVFPEGWDKRYCLDSLDQDSFDTIHFFGNETSPGGNDFEIFADPRTV
HsPMM2      IKLPKKRGTFIEFRNGMLNVSPIGRSCSQEERIEFYELDKKENIRQKFVADLRKEFAGKGLTFSIGGQISFDVFPDGWDKRYCLRHVENDGYKTIYFFGDKTMPGGNDHEIFTDPRTM
            : :* *********.**:*:*****.*: *** :* : ** .::* *:*  *: :*: .* ** ** *******.**** ***  :::  :. *:***:: *  ****.*** . **:

TtPMM-A1         GHTVTSPNDTVQQCKSIFLSE----- 249
TuPMM-A1         GHTVTSPNDTVQQCKSIFLSE----- 249
TaPMM-A1         GHTVTSPNDTVQQCKSIFLSE----- 249
TaPMM-B1         GHTVTSPNDTVQQCKSIFLSE----- 249
TtPMM-B1         GHTVTSPNDTVQQCKSIFLSE----- 249
TaPMM-D1         GHTVTSPNDTVQQCKSIFLSE----- 252
AetPMM-D1        GHTVTSPNDTVQQCKSIFLSE----- 252
HvPMM-1          GHTVTSPNDTVQQCKSIFLSE----- 249
TaPMM-B2         GHTVTSPDDTVQQCRSIFLSK----- 248
TtPMM-B2         GHTVTSPDDTVQHCRSIFLSK----- 248
TaPMM-D2         GHTVTSPDDTVQQCRSIFLSK----- 251
AetPMM-D2        GHTVTSPDDTVQQCRSIFLSK----- 251
BdPMM            GHTVTSPDDTVEQCRSIFLSK----- 249
OsPMM            GHTVTSPDDTAEQCRSLFMSK----- 248
HsPMM1           GHSVVSPQDTVQRCREIFFPETAHEA 262
HsPMM2           GYSVTAPEDTRRIC-ELLFS------ 246
                 *::*.:*:**  . * .:::.
```
