# Supplementary material for: Molecular analysis of phosphomannomutase (PMM) genes reveals a unique PMM duplication event in diverse Triticeae species and the main PMM isozymes in bread wheat tissues
Source: BMC Plant Biol. 2010 Oct 5;10:214. doi: 10.1186/1471-2229-10-214 (PMC3017832; doi:10.1186/1471-2229-10-214)
Supplement: Additional file 4 — Oligonucleotide primers used in this work. [file 1471-2229-10-214-S4.PDF]

Additional file 4: Oligonucleotide primers used in this work

Primer	Sequence (from 5′ to 3′)	Use
PMMf	TTCAGCAGCAGGAGGAAGATG	Amplifying full length cDNA and gDNA of *PMM* genes from A, AB, D and ABD genomes.
PMMr	CGCCAAATGCGCGTATACA	
IFf	CACAAGGACGGCAAGCTCATC	Conserved intron flanking primers. IFf was labeled by D4 fluorescence to facilitate fragment analysis.
IFr	ACATTGATCATTCCACTCCTGAA	
HvPMM-1f	TCCTTCGTAGCAGCTTCAGCAG	Amplifying the cDNA and gDNA of *HvPMM-1*.
HvPMM-1r	TCACTCCGACAGGAAGATGGATTTGC	
HvPMM-2f	TCCTTCCCTGTAGAAGCTCCAGCA	Amplifying the cDNA and gDNA of *HvPMM-2*.
HvPMM-2r	TCGAGGGGGTGTGGAGAACCCA	
BdPMMf	GCTGCTATATCATCATCGTCACG	Amplifying the cDNA and gDNA of *BdPMM*.
BdPMMr	GAAAAGGCAAAAATGTATTGAAGG	
PMM-A1r	GAATTATTTCGAGACGGCAAGT	Specific for *PMM-A1* transcripts.
PMM-B1r	GATCTATAGGGGAATTATTCCGAA	Specific for *PMM-B1* transcripts.
PMM-D1r	TTTGTGAATGTATGTATACCATGTG	Specific for *PMM-D1* transcripts.
PMM-D2r	GAACGAGTTCAAAGGGAGATCT	Specific for *PMM-D2* transcripts.
PMMf1	ACATTCATAGAGTTCAGGAGTGG	Conserved in all *TaPMM* cDNA sequences, pairing with PMM-A1r, PMM-B1r, PMM-D1r and PMM-D2r, respectively, to detect *TaPMM-A1*, *TaPMM-B1*, *TaPMM-D1* or *TaPMM-D2* transcripts in semiquantitative RT-PCR.
PMM-A2f	ATGGTGGCATCGGTGGCAAAC	Amplifying specifically *TaPMM-A2* transcripts in semiquantitative RT-PCR.
PMM-A2r	GGGCTGGTAACTGTATGACC	
PMM-B2f	ATGGCGGCAAAGAAGAAT	Amplifying specifically *TaPMM-B2* transcripts in semiquantitative RT-PCR.
PMM-B2r	AGCTGCTCGGAGATCTTGAC	
Tubulinf1	AGAACACTGTTGTAAGGCTCAAC	Amplifying wheat tubulin transcripts, serving as an internal control in semiquantitative PCR.
Tubulinr1	GAGCTTTACTGCCTCGAACATGG	
PMM-A1-PstIf	AGCTGCAGATGGCGGCGGCGAGG	Construction of p181-A1 for yeast complementation analysis.
PMM-A1-NotIr	AGGCGGCCGCTCACTCCGACAGGAA	
PMM-B1-NotIr	AGGCGGCCGCTCACTCCGAGAGGAA	Pairing with PMM-A1-PstIf to construct p181-B1 for yeast complementation analysis.
PMM-D1-PstIf	AGCTGCAGATGGCGGCGGCGGCG	Pairing with PMM-A1-NotIr to construct p181-D1 for yeast complementation analysis.
PMM-B2-PstIf	AGCTGCAGATGGCGGCAAAGAAGAATGC	Pairing with PMM-D2-NotIr to construct p181-B2 for yeast complementation analysis.
PMM-D2-PstIf	AGCTGCAGATGGTGGCATCAGCGGCG	Construction of p181-D2 for yeast complementation analysis.
PMM-D2-NotIr	TGGCGGCCGCTCACTTTGACAGGAAGATA	
PMM-A1-NdeIf	GGCATATGGCAGCAGCACGAAAGGACGCCGGT	Pairing with TaPMM-A1/D1-SacIr to construct p30a-A1 for bacterial expression.
PMM-D1-NdeIf	GGCATATGGCAGCAGCAGCAGCAGCACGA AAGACCGCCGGT	Pairing with TaPMM-A1/D1-SacIr to construct p30a-D1 for bacterial expression.
TaPMM-A1/D1-SacIr	ACGAGCTCCACTCCGACAGGAAG	
PMM-B1-NdeIf	GGCATATGGCAGCAGCACGAAAGGACGCCGGC	Construction of p30a-B1 for bacterial expression.
PMM-B1-SacIr	ACGAGCTCCACTCCGAGAGGAAGATGGA	

PMM-B2-NdeIf	GGCATATGGCGGCAAAGAAGAATGC	Pairing with PMM-B2/D2-NotIr to construct p30a-B2 for bacterial expression.
PMM-D2-NdeIf	TACATATGGTGGCATCAGCGGCGCGAAAGAACGCCGGGGTG	Pairing with PMM-B2/D2-NotIr to construct p30a-D2 for bacterial expression.
PMM-B2/D2-NotIr	TGGCGGCCGCCTTTGACAGGAAGATAGAT	
OsPMM-BamHIf	TCGGATCCATGGCGGCGAGGAAG	Construction of p30a-OsPMM for bacterial expression.
OsPMM-EcoRIr	AGGAATTCTCACTTCGACATGAA	
HvPMM-NdeIf	GTCATATGGCAGCTGCTGGTAAAAGTACtGGTGTGCTCGCGCTC	Construction of p30a-HvPMM for bacterial expression.
HvPMM-NotIr	CTGCGGCCGCCTCCGACAGGAAGATGG	
BdPMM-NdeIf	GTCATATGGCaGCTGTTAAGAAGAATGCTGGtGTGCTCGCGCTCTTC	Construction of p30a-BdPMM for bacterial expression.
BdPMM-NotIr	CTGCGGCCGCCTTTGACAAGAAGATGG	

The underlined nucleotides form *Bam*HI (GGATCC), *Eco*RI (GAATTC), *Nde*I (CATATG), *Not*I (GCGGCCGC), *Pst*I (CTGCAG), or *Sac*I (GAGCTC) restriction sites.
