# Supplementary material for: Molecular analysis of phosphomannomutase (PMM) genes reveals a unique PMM duplication event in diverse Triticeae species and the main PMM isozymes in bread wheat tissues
Source: BMC Plant Biol. 2010 Oct 5;10:214. doi: 10.1186/1471-2229-10-214 (PMC3017832; doi:10.1186/1471-2229-10-214)

Additional file 5

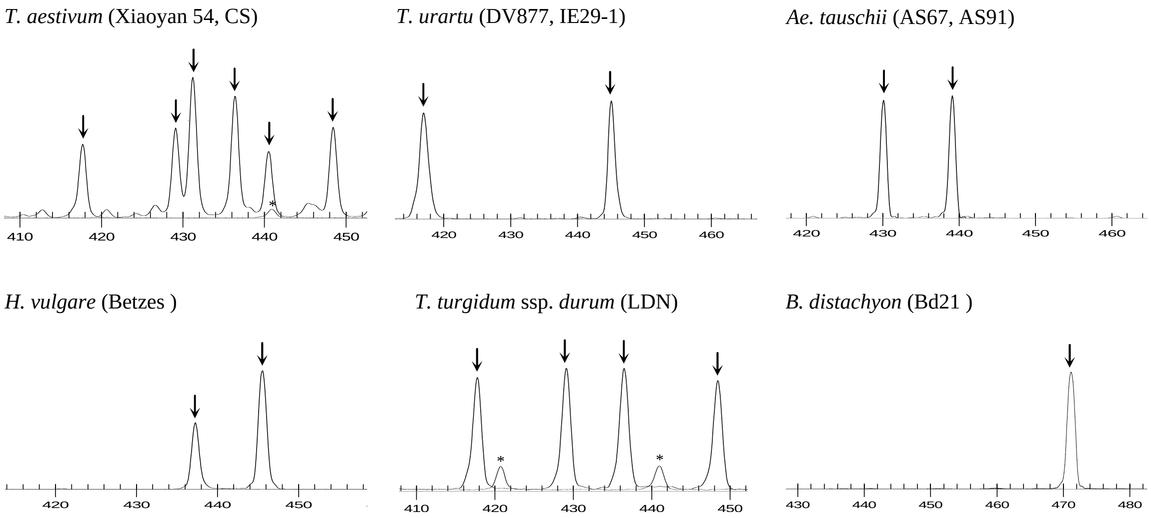

Supplement: Additional file 5 — Investigation of PMM copy numbers in bread wheat and related Triticeae species (T. urartu, Ae. tauschii, H. vulgare, T. turgidum ssp. durum) and B. distachyon by fragment analysis. PMM gene specific peaks are indicated by arrows. The minor peaks labeled by asterisks are caused by DNA size standards. The scale on the horizontal axis indicates fragment size (number of nucleotides), which was determined using the DNA size standards co-separated with the PCR products. The specific genotypes used in this analysis are provided in the brackets. The data shown are representative of five independent experiments. [file 1471-2229-10-214-S5.PDF]
