# Supplementary material for: Molecular analysis of phosphomannomutase (PMM) genes reveals a unique PMM duplication event in diverse Triticeae species and the main PMM isozymes in bread wheat tissues
Source: BMC Plant Biol. 2010 Oct 5;10:214. doi: 10.1186/1471-2229-10-214 (PMC3017832; doi:10.1186/1471-2229-10-214)
Supplement: Additional file 6 — Estimation of PMM copy numbers in the diploid and tetraploid species possessing additional Triticeae genomes. [file 1471-2229-10-214-S6.PDF]

Additional file 6: Estimation of *PMM* copy numbers in the diploid and tetraploid species possessing additional *Triticeae* genomes

Ploidy level	Species	Genome	Number of *PMM* specific fragment	Germplasm lines used during the analysis
Diploid (2n = 14)	*T. monococcum*	A^mA^m	2	TA2024, TA2026, TA2030, TA2034, TA2714, TA2715, TA2722, TA2725
	Ae. markgrafii	CC	2	PI203431, PI 263554, PI 542198, PI 542200, PI 542206, PI 542209
	Ae. comosa	MM	2	PI 551018, PI 551022, PI 551024, PI 542172, PI 542174, PI 542175, PI 542176
	Ae. uniaristata	NN	2	PI 276995, PI 276996, PI 374326, PI 554418, PI 554419, PI 554420, PI 554421
	Secale cereale	RR	2	PI 29447, PI 323376, PI 446244, PI 534935
	Ae speltoides	SS	2	PI 449338, PI 486263, PI 554295, KU-2-1, PI 173614
	Ae. bicornis	S^bS^b	2	CIae47, KU-14610-1, KU-14615-1
	Ae. longissima	S^1S^1	2	KU-14621-1, KU-14627-1, PI 604129, PI 604131, PI 604142
	Ae. searsii	S^sS^s	2	KU-14652-1, KU-14656-1, PI 599157, PI 599160, PI 599177
	Ae. sharonensis	$S^{sh}S^{sh}$	2	PI 542237, PI 584370, PI 584379, PI 584392, PI 584346
	Pseudoroegneria stipifolia	S^tS^t	2	PI 636641
	Ae. umbellulata	UU	2	PI 486260, PI 542364, PI 542369, KU-12199
Tetraploid (2n = 28)	*T. timopheevii*	AAGG	4	PI 190974, PI 286073, PI 266850, PI 282932, PI 288033, PI 290518, KU-107-1, KU-107-2
	Ae. cylindrica	DDCC	4	PI 172357, PI 254864, PI 276977, PI 499259
	Ae. crassa	DDMM	4	PI 227434, PI 487286, PI 574459, KU-21-4, KU-21-6
	Ae. ventricosa	DDNN	4	KU-12966
	Ae. biuncialis	UUMM	4	PI 614606, PI 614608, PI 614610, PI 614612, KU-12790, KU-11488
	Ae. columnaris	UUMM	4	KU-11-1, KU-11-3
	Ae. geniculata	UUMM	4	PI 170210, PI 388754, PI 487220, PI 487283, PI 491429, PI 614625
	Ae. ovata	UUMM	4	KU-12417

The TA accessions are from Wheat Genetic & Genomic Resources Center, Kansas State University, USA. The CIae and PI accessions are from USDA-ARE National Small Grains Collection, Aberdeen, USA. The KU accessions are from the National BioResource Project-wheat, Japan.
