# Supplementary figures and images for: Molecular analysis of phosphomannomutase (PMM) genes reveals a unique PMM duplication event in diverse Triticeae species and the main PMM isozymes in bread wheat tissues
Source: BMC Plant Biol. 2010 Oct 5;10:214. doi: 10.1186/1471-2229-10-214 (PMC3017832; doi:10.1186/1471-2229-10-214)

# Additional file 7

(a)

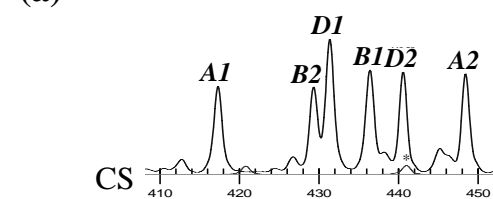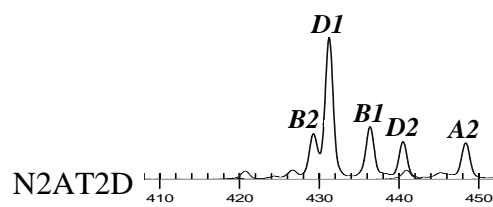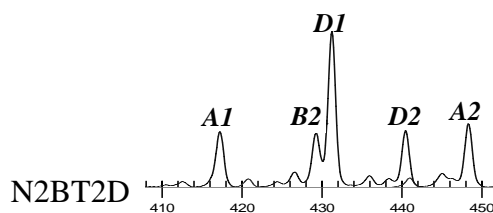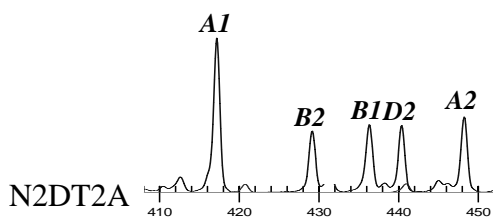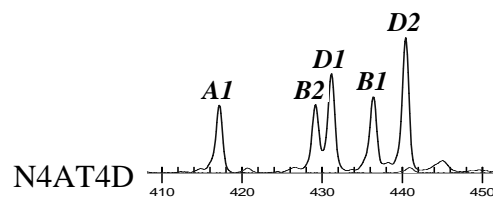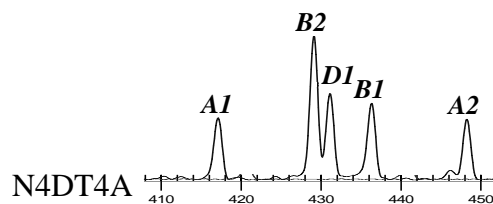

(b)

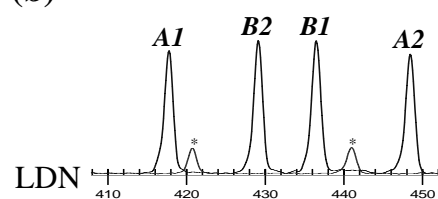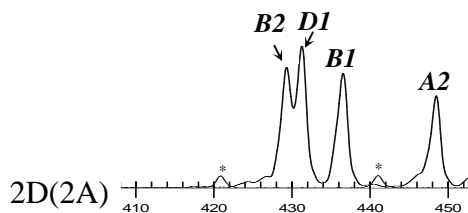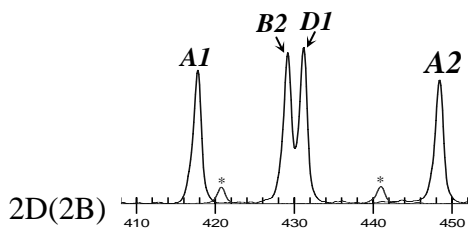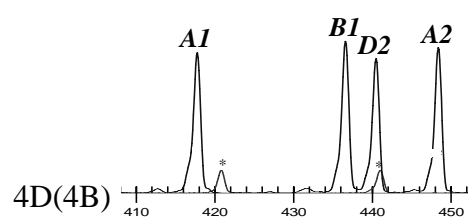

Supplement: Additional file 7 — Chromosomal assignment of PMM genes in hexaploid and tetraploid wheats. The fragments specific for individual PMM gene members were amplified by PCR, followed by separation via capillary electrophoresis. The templates for PCR were prepared from Chinese Spring (CS), the nulli-tetrasomic (NT) lines of CS, the durum wheat variety Langdon (LDN), and the D genome substitution lines in LDN background. The PMM genes represented by the major peaks (fragments) are shown. The minor peaks labeled by asterisks are due to DNA size markers. The scale on the horizontal axis indicates fragment size. (a) Compared to the presence of all six PMM specific fragments in CS, the fragments representing PMM-A1, B1, D1, A2 or D2 genes were specifically absent from the NT lines lacking chromosomes 2A (N2AT2D), 2B (N2BT2D), 2D (N2DT2A), 4A (N4AT4D) or 4D (N4DT4A). The six PMM specific fragments were all amplified in the NT lines lacking other groups (i.e., 1, 3, 5, 6 and 7) of chromosomes (data not shown). (b) The presence of four PMM specific fragments in LDN. Note that the fragments representing PMM-A1, B1 or B2 genes were not amplified from the substitution lines 2D(2A) (lacking 2A chromosome), 2D(2B) (lacking 2B chromosome) or 4D(4B) (lacking 4B chromosome). The fragment representing PMM-D1 was amplified from 2D(2A) and 2D(2B) owing to the presence of 2D chromosome in the two lines. Similarly, the fragment representing PMM-D2 was amplifiable from 4D(4B) due to the presence of 4D chromosome in this line. The PMM-A1, B1, A2 and B2 fragments were all amplifiable in the other substitution lines in which 2A, 2B, 4A and 4B chromosomes are not involved in the substitution by D genome chromosomes (data not shown). The data displayed are typical of five independent sets of experiments. [file 1471-2229-10-214-S7.PDF]

Additional file 8

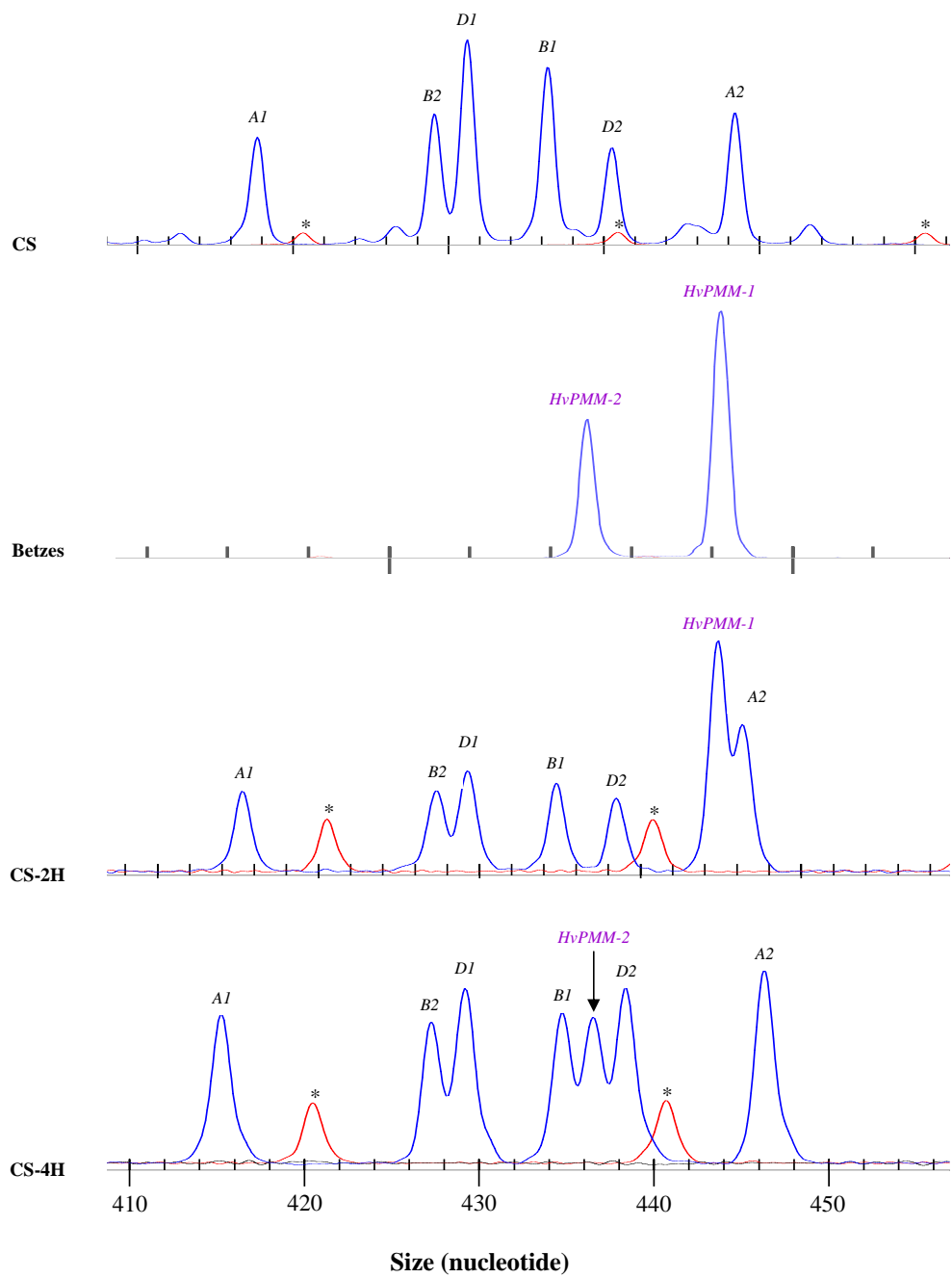

Supplement: Additional file 8 — Chromosomal locations of barley PMM genes (HvPMM-1 and 2). The genomic DNA samples, extracted from the bread wheat variety Chinese Spring (CS), barley variety Betzes, and barley chromosome addition lines in CS background, were used for PCR amplifications with the nucleotide primers capable of recognizing PMM genes from Triticeae species. The peaks in blue represent PCR fragments amplified from specific bread wheat or barley PMM genes. The six PMM genes in CS are abbreviated as A1, B1, D1, A2, B2 and D2, respectively. HvPMM-1 and 2 were found in the two addition lines containing barley 2H (CS-2H) and 4H (CS-4H) chromosomes, respectively. The fragment pattern of the remaining addition lines was identical to that of CS (data not shown). The peaks marked by asterisks were caused by DNA size standards. The data shown are typical of three independent experiments. [file 1471-2229-10-214-S8.PDF]

## Additional file 9

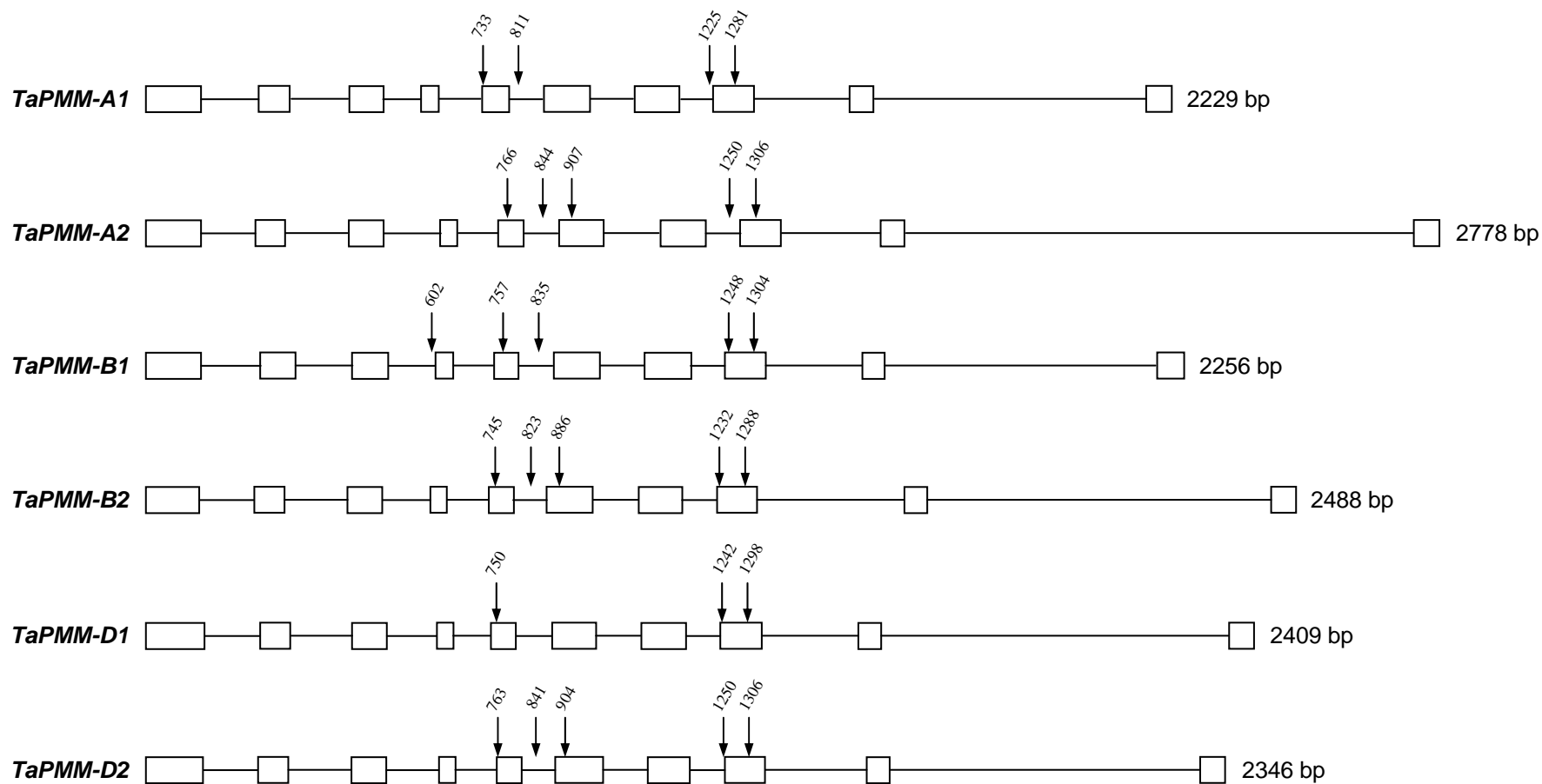

500 bp

Supplement: Additional file 9 — Distribution of multiple EcoRI sites in the genomic ORFs of six bread wheat PMM genes. The locations of the sites are calculated in relation to the start codon ATG (with the A nucleotide as 1). The exons are represented by boxes. [file 1471-2229-10-214-S9.PDF]

## Additional file 11

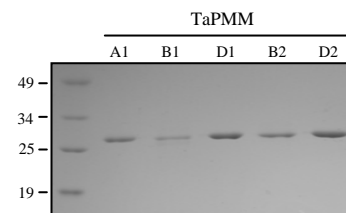

Supplement: Additional file 11 — SDS-PAGE analysis of recombinant TaPMM-A1, B1, D1, B2, and D2 proteins purified using metal chelate affinity chromatography. The size of protein molecular mass (kD) standards is shown on the left side of the graph. [file 1471-2229-10-214-S11.PDF]
