# Supplementary material for: Molecular analysis of phosphomannomutase (PMM) genes reveals a unique PMM duplication event in diverse Triticeae species and the main PMM isozymes in bread wheat tissues
Source: BMC Plant Biol. 2010 Oct 5;10:214. doi: 10.1186/1471-2229-10-214 (PMC3017832; doi:10.1186/1471-2229-10-214)

Additional file 10

(a)

```
TaPMM-D1    ATG GCG GCG GCG GCG GCG GCG AGG AAG ACC GCC GGT GTG CTC GCG CTC TTC GAC GTC GAC GGC ACC CTC ACC GCG CCC CGC AAG GAG GTG ACG CCG GAG ATG CTC GAG TTC ATG AAG CGC
AetPMM-D1   ATG GCG GCG GCG GCG GCG GCG AGG AAG ACC GCC GGT GTG CTC GCG CTC TTC GAC GTC GAC GGC ACC CTC ACC GCG CCC CGC AAG GAG GTG ACG CCG GAG ATG CTC GAA TTC ATG AAG CGC
TaPMM-B1    ATG GCG GCG GCG --- --- --- AGG AAG GAC GCC GGC GTG CTC GCG CTC TTC GAC GTC GAC GGC ACC CTC ACC GCG GCC CGC AAG GAG GTG ACG CCG GAG ATG CTC GAG TTC ATG AAG CGC
TtPMM-B1    ATG GCG GCG GCG --- --- --- AGG AAG AAC GCC GGC GTG CTC GCG CTC TTC GAC GTC GAC GGC ACC CTC ACC GCG GCC CGC AAG GAG GTG ACG CCG GAG ATG CTC GAG TTC ATG AAG CGC
TaPMM-A1    ATG GCG GCG GCG --- --- --- AGG AAG GAC GCC GGT GTG CTC GCG CTC TTC GAC GTC GAC GGC ACC CTC ACC GCG CCC CGC AAG GAG GTG ACG CCG GAG ATG CTC GAG TTC ATG AAG CGC
TtPMM-A1    ATG GCG GCG GCG --- --- --- AGG AAG GAC GCC GGT GTG CTC GCG CTC TTC GAC GTC GAC GGC ACC CTC ACC GCG CCC CGC AAG GAG GTG ACG CCG GAG ATG CTC GAG TTC ATG AAG CGC
TuPMM-A1    ATG GCG GCG GCG --- --- --- AGG AAG GAC GCC GGT GTG CTC GCG CTC TTC GAC GTC GAC GGC ACC CTC ACC GCG CCC CGC AAG GAG GTG ACG CCG GAG ATG CTC GAG TTC ATG AAG CGC
TaPMM-B2    ATG GCG GCA --- --- --- --- AAG AAG AAT GCC GGG GTG CTC GCA CTC TTC GAC GTC GAC GGC ACC CTC ACC GCC CCC TGC AAG GAG GTG ACG CCG GAG TTG CTC GAG TTT ATG AAG CGC
TtPMM-B2    ATG GCG GCA --- --- --- --- AAG AAG AAT GCC GGG GTG CTC GCA CTC TTC GAC GTC GAC GGC ACC CTC ACC GCC CCC CGC AAG GAG GTG ACG CCG GAG TTG CTC GAG TTT ATG AAG CGC
TaPMM-D2    ATG GTG GCA TCA GCG GCG --- AGG AAG AAC GCC GGG GTG CTC GCG CTC TTC GAC GTC GAT GGC ACC CTC ACC GCC CCC CGC AAG GAG GTG ACG CCG GAG ATG CTC GAG TTC ATG AAG CGC
AetPMM-D2   ATG GTG GCA TCA GCG GCG --- AGG AAG AAC GCC GGG GTG CTC GCG CTC TTC GAC GTC GAT GGC ACC CTC ACC GCC CCC CGC AAG GAG GTG ACG CCG GAG ATG CTC GAG TTC ATG AAG CGC

CTG CGC GAG AAT GTG ACC GTC GGC GTG GTG GGG GGA TCC GAT TTG GTC AAG ATC TCC GAG CAG CTC GGC AAA TCA GTT ATC ACC GAC TAC GAC TAC GTC TTC TCC GAG AAT GGC CTG GTC GCT CAC AAG
CTG CGC GAG AAT GTG ACC GTC GGC GTG GTG GGG GGA TCC GAT CTG GTC AAG ATC TCC GAG CAG CTC GGC AAA TCA GTT ATC ACC GAC TAC GAC TAC GTC TTC TCC GAG AAT GGC CTG GTC GCT CAC AAG
CTG CGC GAG AAT GTG ACC GTC GGC GTG GTG GGG GGA TCC GAT CTG GTC AAG ATC TCC GAG CAG CTC GGC AAA TCA GTC ATC ACC GAC TAC GAC TAC GTC TTC TCC GAG AAC GGC CTG GTC GCG CAC AAG
CTG CGC GAG AAT GTG ACC GTC GGC GTG GTG GGG GGA TCC GAT CTG GTC AAG ATC TCC GAG CAG CTC GGC AAA TCA GTC ATC ACC GAC TAC GAC TAC GTC TTC TCC GAG AAC GGC CTG GTC GCG CAC AAG
CTG CGC GAG AAT GTG ACC GTC GGC GTG GTG GGG GGA TCC GAT CTG GTC AAG ATC TCC GAG CAG CTC GGC AAA TCA GTC ATC ACC GAC TAT GAC TAC GTC TTC TCC GAG AAC GGC CTG GTC GCG CAC AAG
CTG CGC GAG AAT GTG ACC GTC GGC GTG GTG GGG GGA TCC GAT CTG GTC AAG ATC TCC GAG CAG CTC GGC AAA TCA GTC ATC ACC GAC TAT GAC TAC GTC TTC TCC GAG AAC GGC CTG GTC GCG CAC AAG
CTG CGC GAG AAT GTG ACC GTC GGC GTG GTG GGG GGA TCC GAT CTG GTC AAG ATC TCC GAG CAG CTC GGC AAA TCA GTC ATC ACC GAC TAT GAC TAC GTC TTC TCC GAG AAC GGC CTG GTC GCG CAC AAG
CTG CGT GAG AAT GTG ACC GTC GGC GTG GTG GGG GGA TCC GAT CTG GTC AAG ATC TCC GAG CAG CTT GCC AAA TCA GTT ATC ACC GAC TAT GAC TAC GTC TTC TCC GAG AAC GGC CTG GTC GCG CAC AAG
CTG CGT GAG AAT GTG ACC GTC GGC GTG GTG GGG GGA TCC GAT CTG GTC AAG ATC TCC GAG CAG CTT GCC AAA TCA GTT ATC ACC GAC TAT GAC TAC GTC TTC TCC GAG AAC GGC CTG GTC GCG CAC AAG
CTG CGT GAG AAT GTG ACC GTC GGT GTG GTG GGG GGA TCC GAT CTG GTC AAG ATC TCC GAG CAG CTC GGC AAA TCA GTT ATC ACC GAC TAT GAC TAC GTC TTC TCC GAG AAT GGC CTG GTC GCG CAC AAG
CTG CGT GAG AAT GTG ACC GTC GGT GTG GTG GGG GGA TCC GAT CTG GTC AAG ATC TCC GAG CAG CTC GGC AAA TCA GTT ATC ACC GAC TAT GAC TAC GTC TTC TCC GAG AAT GGC CTG GTC GCG CAC AAG

GAC GGC AAG CTC ATC GGG ACG CAA AGC TTG AAA ACG TAT CTT GGA GAT GAC CAG CTT AAG GAA TTC ATT AAC TTC ACT CTT CAT TAC ATT GCG GAC TTG GAT ATC CCA ATT AAA AGG GGT ACA TTC ATA
GAC GGC AAG CTC ATC GGG ACG CAA AGC TTG AAA ACG TAT CTT GGA GAT GAC CAG CTT AAG GAA TTC ATT AAC TTC ACT CTT CAT TAC ATT GCG GAC TTG GAT ATC CCA ATT AAA AGG GGT ACA TTC ATA
GAC GGC AAG CTC ATC GGG ACG CAA AGC TTG AAA ACG TAT CTT GGA GAT GAC CAG CTT AAG GAA TTC ATT AAC TTC ACT CTT CAT TAC ATT GCG GAC TTG GAT ATC CCA ATT AAA AGG GGT ACA TTC ATA
GAC GGC AAG CTC ATC GGG ACG CAA AGC TTG AAA ACG TTT CTT GGA GAT GAC CAG CTT AAG GAA TTC ATT AAC TTC ACT CTT CAT TAC ATT GCG GAC TTG GAT ATC CCA ATT AAA AGG GGT ACA TTC ATA
GAC GGC AAG CTC ATC GGG ACG CAA AGC TTG AAA ACG TAT CTT GGA GAT GAC CAG CTT AAG GAA TTC ATT AAC TTC ACT CTT CAT TAC ATC GCG GAT TTG GAT ATC CCA ATT AAA AGG GGT ACA TTC ATA
GAC GGC AAG CTC ATC GGG ACG CAA AGC TTG AAA ACG TAT CTT GGA GAT GAC CAG CTT AAG GAA TTC ATT AAC TTC ACT CTT CAT TAC ATC GCG GAT TTG GAT ATC CCA ATT AAA AGG GGT ACA TTC ATA
GAC GGC AAG CTC ATC GGG ACG CAA AGC TTG AAA ACG TAT CTT GGA GAT GAC CAA CTT AAG GAA TTC ATT AAC TTC ACT CTT CAT TAC ATC GCG GAT TTG GAT ATC CCA ATT AAA AGG GGT ACA TTC ATA
GAC GGC AAG CTC ATC GGG ACA CAA AGC TTG AAA ACG CAT CTT GGA GAT GAC CAG CTT AAG GAA TTC ATT AAC TTC ACC CTT CAT TAC ATT GCG GAT TTG GAT ATC CCA ATT AAA AGA GGC ACA TTC ATA
GAC GGC AAG CTC ATC GAG ACA CAA AGC TTG AAA ACG CAT CTT GGA GAT GAC CAG CTT AAG GAA TTC ATT AAC TTC ACC CTT CAT TAC ATT GCG GAT TTG GAT ATC CCA ATT AAA AGA GGC ACA TTC ATA
GAC GGC AAG CTC ATC GGG ACG CAA AGC TTG AAA ACG TAT CTT GGA GAT GAC CAG CTT AAG GAA TTC ATT AAC TTC ACT CTT CAT TAC ATT GCG GAT TTG GAT ATC CCA ATT AAA AGA GGC ACA TTC ATA
GAC GGC AAG CTC ATC GGG ACG CAA AGC TTG AAA ACG TAT CTT GGA GAT GAC CAG CTT AAG GAA TTC ATT AAC TTC ACT CTT CAT TAC ATT GCG GAT TTG GAT ATC CCA ATT AAA AGA GGC ACA TTC ATA

GAG TTC AGG AGT GGA ATG ATC AAT GTG TCG CCT ATA GGG AGG AAC TGT AGT CAA GAA GAA CGT GAT GAT TTT GAG AAG TAT GAT AAG GTA CAT AAC GTT CGG CCT AAA ATG GTG TCA GTG CTT CGC GAA
GAG TTC AGG AGT GGA ATG ATC AAT GTG TCG CCT ATA GGG AGG AAC TGT AGT CAA GAA GAA CGT GAT GAT TTT GAG AAG TAT GAT AAG GTA CAT AAC GTT CGG CCT AAA ATG GTG TCA GTG CTT CGC GAA
GAG TTC AGG AGT GGA ATG ATC AAT GTG TCG CCC ATA GGG AGG AAC TGT AGT CAG GAA GAA CGT GAT GAT TTT GAG AAG TAT GAT AAG GTA CAT AAC GTT CGG CCT AAA ATG GTG TCA GTG CTT CGC GAA
GAG TTC AGG AGT GGA ATG ATC AAT GTG TCG CCC ATA GGG AGG AAC TGT AGT CAG GAA GAA CGT GAT GAT TTT GAG AAG TAT GAT AAG GTA CAT AAC GTT CGG CCT AAA ATG GTG TCA GTG CTT CGC GAA
GAG TTC AGG AGT GGA ATG ATC AAT GTG TCG CCT ATA GGG AGG AAC TGT AGT CAA GAA GAA CGT GAT GAT TTT GAG AAG TAT GAT AAG GTA CAT AAC GTT CGG CCT AAA ATG GTG TCA GTG CTT CGT GAA
GAG TTC AGG AGT GGA ATG ATC AAT GTG TCG CCT ATA GGG AGG AAC TGT AGT CAA GAA GAA CGT GAT GAT TTT GAG AAG TAT GAT AAG GTA CAT AAC GTT CGG CCT AAA ATG GTG TCA GTG CTT CGT GAA
GAG TTC AGG AGT GGA ATG ATC AAT GTG TCG CCT ATA GGG AGG AAC TGT AGT CAA GAA GAA CGT GAT GAT TTT GAG AAG TAT GAT AAG GTA CAT AAC GTT CGG CCT AAA ATG GTG TCA GTG CTT CGT GAA
GAA TTC AGG AGT GGA ATG ATC AAT GTG TCG CCT ATA GGG AGG AAC TGT AGT CAA GAA GAA CGT GAT GAT TTT GAG AAG TAT GAT AAG GTA CAT AAC GTT CGG CCT AAA ATG GTG TCA GTG CTT CGT GAA
GAA TTC AGG AGT GGA ATG ATC AAT GTG TCG CCT ATA GGG AGG AAC TGT AGT CAA GAA GAA CGT GAT GAT TTT GAG AAG TAT GAT AAG GTA CAT AAC GTT CGG CCT AAA ATG GTG TCA GTG CTT CGT GAA
GAA TTC AGG AGT GGA ATG ATC AAT GTG TCG CCT ATA GGG AGG AAC TGT AGT CAA GAA GAA CGT GAT GAT TTT GAG AAG TAT GAT AAG GTA CAT AAC GTT CGG CCT AAA ATG GTG TCA ATG CTT CGT GAA
GAA TTC AGG AGT GGA ATG ATC AAT GTG TCG CCT ATA GGG AGG AAC TGT AGT CAA GAA GAA CGT GAT GAT TTT GAG AAG TAT GAT AAG GTA CAT AAC GTT CGG CCT AAA ATG GTG TCA ATG CTT CGT GAA

AAG TTT GCA CAC CTG AAC CTG ACC TTT TCT ATT GGA GGG CAG ATC AGT TTT GAT GTA TTC CCG CAA GGC TGG GAC AAA ACC TAC TGC TTG AGA TAT CTC GAA GAA TTC AAA GAA ATC CAT TTC TTT GGG
AAG TTT GCA CAC CTG AAC CTG ACC TTT TCT ATT GGA GGG CAG ATC AGT TTT GAT GTA TTC CCG CAA GGC TGG GAC AAA ACC TAC TGC TTG AGA TAT CTC GAA GAA TTC AAA GAA ATC CAT TTC TTT GGG
AAG TTT GCA CAC CTG AAC CTG ACT TTT TCC ATT GGA GGG CAG ATC AGT TTT GAT GTA TTC CCA CAA GGC TGG GAC AAA ACC TAC TGC TTG AGA TAT CTG GAA GAA TTC AAG GAA ATC CAT TTC TTT GGG
AAG TTT GCA CAC CTG AAC CTG ACT TTT TCC ATT GGA GGG CAG ATC AGT TTT GAT GTA TTC CCA CAA GGC TGG GAC AAA ACC TAC TGC TTG AGA TAT CTG GAA GAA TTC AAG GAA ATC CAT TTC TTT GGG
AAG TTT GCA CAC CTG AAC CTG ACT TTT TCT ATT GGA GGG CAG ATC AGT TTT GAT GTA TTC CCA CAA GGC TGG GAC AAA ACC TAC TGC TTG AGA TAT CTG GAA GAA TTC AAA GAA ATC CAT TTC TTT GGG
AAG TTT ACA CAC CTG AAC CTG ACT TTT TCT ATT GGA GGA CAG ATC AGT TTT GAT GTA TTC CCA CAA GGC TGG GAC AAA ACC TAC TGC TTG AGA TAT CTG GAA GAA TTC AAA GAA ATC CAT TTC TTT GGG
AAG TTT GCA CAC CTG AAC CTG ACT TTT TCT ATT GGA GGG CAG ATC AGT TTT GAT GTA TTC CCA CAA GGC TGG GAC AAA ACC TAC TGC TTG AGA TAT CTG GAA GAA TTC AAA GAA ATC CAT TTC TTT GGG
AAG TTT GCA CAC CTG AAC CTG ACT TTT TCT ATT GGA GGG CAG ATC AGT TTT GAT GTA TTC CCA CAT GGC TGG GAC AAA ACC TAC TGC TTG AGA TAT CTC GAC GAA TTC AAA GAA ATT CAT TTC TTT GGG
AAG TTT GCA CAC CTG AAC CTG ACT TTT TCT ATT GGA GGG CAG ATC AGT TTT GAT GTA TTC CCA CAT GGC TGG GAC AAA ACC TAC TGC TTG AGA TAT CTC GAC GAA TTC AAA GAA ATT CAT TTC TTT GGG
AAG TTT GCA CAC CTG AAC CTG ACT TTT TCT ATT GGA GGG CAG ATC AGT TTT GAT GTA TTC CCA CAT GGC TGG GAC AAA ACC TAC TGC TTG AGA TAT CTC GAC GAA TTC AAA GAA ATT CAT TTC TTT GGG
AAG TTT GCA CAC CTG AAC CTG ACT TTT TCT ATT GGA GGG CAG ATC AGT TTT GAT GTA TTT CCA CAT GGC TGG GAC AAA ACC TAC TGC TTG AGA TAT CTC GAC GAA TTC AAA GAA ATT CAT TTC TTT GGG

GAC AAA ACC TAC AAG GGT GGC AAT GAT CAT GAG ATA TTT GAA TCT GAA AGA ACA GTT GGT CAT ACA GTT ACC AGC CCC AAT GAC ACG GTG CAG CAG TGC AAA TCC ATC TTC CTG TCG GAG     756
GAC AAA ACC TAC AAG GGT GGC AAT GAT CAT GAG ATA TTT GAA TCT GAA AGA ACA GTT GGT CAT ACA GTT ACC AGC CCC AAT GAC ACG GTG CAG CAG TGC AAA TCC ATC TTC CTG TCG GAG     756
GAC AAA ACC TAC AAG GGT GGC AAT GAT CAT GAG ATA TTT GAA TCT GAC AGA ACA GTT GGT CAT ACA GTT ACC AGC CCC AAT GAC ACG GTG CAG CAG TGC AAA TCC ATC TTC CTC TCG GAG     747
GAC AAA ACC TAC AAG GGT GGC AAT GAT CAT GAG ATA TTT GAA TCT GAC AGA ACA GTT GGT CAT ACA GTT ACC AGC CCC AAT GAC ACG GTG CAG CAG TGC AAA TCC ATC TTC CTC TCG GAG     747
GAC AAA ACC TAC AAG GGT GGC AAT GAT CAT GAG ATA TTT GAA TCT GAC AGA ACA GTT GGT CAT ACA GTT ACC AGC CCC AAT GAC ACT GTG CAG CAG TGC AAA TCC ATC TTC CTG TCG GAG     747
GAC AAA ACC TAC AAG GGT GGC AAT GAT CAT GAG ATA TTT GAA TCT GAC AGA ACA GTT GGT CAT ACA GTT ACC AGC CCC AAT GAC ACT GTG CAG CAG TGC AAA TCC ATC TTC CTG TCG GAG     747
GAC AAA ACC TAC AAG GGT GGC AAT GAT CAT GAG ATA TTT GAA TCT GAC AGA ACA GTT GGT CAT ACA GTT ACC AGC CCC AAT GAC ACT GTG CAG CAG TGC AAA TCC ATC TTC CTG TCG GAG     747
GAC AAA ACC TAC AAG GGT GGC AAT GAT CAT GAG ATA TTT GAA TCT GAC AGA ACA GTT GGT CAT ACA GTT ACC AGC CCC GAC GAC ACG GTG CAG CAG TGC AGA TCT ATC TTC CTG TCA AAG     744
GAC AAA ACC TAC AAG GGT GGC AAT GAT CAT GAG ATA TTT GAA TCT GAC AGA ACA GTT GGT CAT ACA GTT ACC AGC CCC GAC GAC ACG GTG CAG CAC TGC AGA TCT ATC TTC CTG TCA AAG     744
GAC AAA ACC TAC AAG GGT GGC AAT GAT CAT GAG ATA TTT GAA TCT GAC AGA ACA GTT GGT CAT ACT GTC ACC AGC CCT GAT GAC ACA GTG CAG CAG TGC AGA TCT ATC TTC CTG TCA AAG     753
GAC AAA ACC TAC AAG GGT GGC AAT GAT CAT GAG ATA TTT GAA TCT GAC AGA ACA GTT GGT CAT ACT GTC ACC AGC CCT GAT GAC ACA GTG CAG CAG TGC AGA TCT ATC TTC CTG TCA AAG     753
```

Additional file 10

(b)

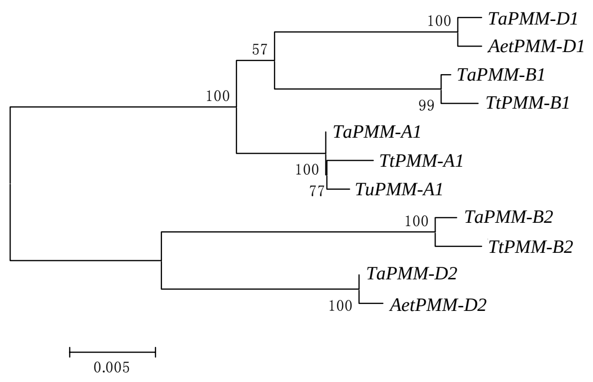

Supplement: Additional file 10 — The cDNA sequence alignment and phylogenetic genetic tree of PMM genes used for evolutionary rate and positive selection analysis. (a) Multiple alignment of the cDNA sequences of 11 active PMM genes from bread wheat and its progenitor species. The sequences are each presented in codon format. The stop codon is not included in the alignment. Codon site 10 (numbered according to TaPMM-D1), which was significantly positively selected based on based on NEB posterior probability, is labeled in purple. (b) The phylogenetic tree used for evolutionary analysis. This tree was constructed using neighbor joining program with complete deletion and the Kimura-2 nucleotide substitution model. The bootstrap value was estimated using 500 replications. [file 1471-2229-10-214-S10.PDF]
