# Supplementary material for: Molecular analysis of phosphomannomutase (PMM) genes reveals a unique PMM duplication event in diverse Triticeae species and the main PMM isozymes in bread wheat tissues
Source: BMC Plant Biol. 2010 Oct 5;10:214. doi: 10.1186/1471-2229-10-214 (PMC3017832; doi:10.1186/1471-2229-10-214)
Supplement: Additional file 12 — GenBank accession numbers of the PMM genes isolated in this work. [file 1471-2229-10-214-S12.PDF]

Additional file 12: GenBank accession numbers of the *PMM* genes isolated in this work

Species	Gene	Accession number
Triticum aestivum	*TaPMM-A1*	GQ412259
	TaPMM-A2	GQ412260
	TaPMM-B1	GQ412261
	TaPMM-B2	GQ412262
	TaPMM-D1	GQ412263
	TaPMM-D2	GQ412264
Triticum turgidum	*TtPMM-A1*	GQ412265
	TtPMM-A2	GQ412266
	TtPMM-B1	GQ412267
	TtPMM-B2	GQ412268
Triticum urartu	*TuPMM-A1*	GQ412269
	TuPMM-A2	GQ412270
Aegilops tauschii	*AetPMM-D1*	GQ412271
	AetPMM-D2	GQ412272
Hordeum vulgare	*HvPMM-1*	GQ412273
	HvPMM-2	GQ412274
Brachypodium distachyon	*BdPMM*	GQ412275
